# *Lactobacillus acidophilus* DDS-1 Modulates the Gut Microbiota and Improves Metabolic Profiles in Aging Mice

**DOI:** 10.3390/nu10091255

**Published:** 2018-09-06

**Authors:** Ravichandra Vemuri, Tanvi Shinde, Rohit Gundamaraju, Shakuntla V. Gondalia, Avinash V. Karpe, David J. Beale, Christopher J. Martoni, Rajaraman Eri

**Affiliations:** 1 School of Health Sciences, College of Health and Medicine, University of Tasmania, Launceston, Tasmania 7250, Australia; tanvi.shinde@utas.edu.au (T.S.); rohit.gundamaraju@utas.edu.au (R.G.); 2 Centre for Food Safety and Innovation, Tasmanian Institute of Agriculture, University of Tasmania, Launceston, Tasmania 7250, Australia; 3 Centre for Human Psychopharmacology, Swinburne University of Technology, Hawthorn, Victoria 3122, Australia; s.gondalia@swin.edu.au; 4 CSIRO Land and Water, Ecosciences Precinct, Dutton Park 4102, Queensland 2601, Australia; avinash.karpe@csiro.au (A.V.K.); david.beale@csiro.au (D.J.B.); 5 UAS Laboratories, Madison, WI, 54401, USA; cmartoni@uaslabs.com

**Keywords:** aging, gut microbiota, metabolism, probiotics

## Abstract

Recent evidence suggests that gut microbiota shifts can alter host metabolism even during healthy aging. *Lactobacillus acidophilus* DDS-1, a probiotic strain, has shown promising probiotic character in vitro, as well as in clinical studies. The present study was carried out to investigate whether DDS-1 can modulate the host metabolic phenotype under the condition of age-affected gut microbial shifts in young and aging C57BL/6J mice. Collected fecal samples were analyzed using 16S rRNA gene sequencing for identifying gut microbiota and untargeted gas chromatography-mass spectrometry (GC-MS) metabolomics analysis. Gut microbial shifts were observed in the control groups (young and aging), leading to an alteration in metabolism. Principal coordinate analysis (PCoA) of microbiota indicated distinct separation in both the DDS-1-treated groups. *L. acidophilus* DDS-1 increased the relative abundances of beneficial bacteria, such as *Akkermansia muciniphila* and *Lactobacillus* spp., and reduced the relative levels of opportunistic bacteria such as *Proteobacteria* spp. Metabolic pathway analysis identified 10 key pathways involving amino acid metabolism, protein synthesis and metabolism, carbohydrate metabolism, and butanoate metabolism. These findings suggest that modulation of gut microbiota by DDS-1 results in improvement of metabolic phenotype in the aging mice.

## 1. Introduction

Microbes residing in a mammalian gut co-evolve with age. These microbes, known as the ‘gut microbiota’, interact with each other, as well as the host, and impact the health of the host [1]. Alterations in gut microbial communities are known to not only influence but also act as a possible causative factor for various gastrointestinal (GI) and metabolic disorders [1,2,3]. Apart from antibiotics, diet, and environmental factors, the age of the host is also linked with the gut microbial changes [2,4,5]. Recently, human and animal studies have reported microbial shifts in aging gut under healthy conditions [1,2,3,5]. From these studies, it is known that *Bacteroidetes* and *Firmicutes*, which dominate in younger individuals, are being reduced with the age [2,4,6]. In addition, the abundance of beneficial bacteria, such as *Akkermansia* spp. and *Lactobacillus* spp., were reduced in the aging population [7,8,9]. Such drastic microbial shifts in healthy-aging could lead to increased intestinal inflammation and changes in host metabolism.

Along these lines, a few animal and clinical studies demonstrated metabolic trajectory associated with gut microbiome upon aging [10,11,12,13]. Primarily, these studies indicated alterations in levels of metabolites involved in the metabolism of amino acids, carbohydrates, nucleotides, lipid, and short-chain fatty acids (SCFA) in the aging group compared to the young group, leading from normal to putrefactive metabolism [10,14,15]. Given the potential for gut microbiota to change with age and its influence on host metabolism, modulation of gut microbial composition offers an opportunity to promote digestive health among the aging population. A number of studies have demonstrated that the lactic acid bacteria (LAB) probiotic strains, especially *Lactobacillus* spp., are natural, safe, and beneficial modulators of the gut microbial composition and metabolic phenotype [16,17,18]. Indeed, clinical studies have shown that supplementation of *Lactobacillus* strains improved the overall health in elderly individuals [17,19,20,21,22,23].

Previously, we showed that DDS-1 strain has a promising ability to survive under stressed conditions (pH, salts, and digestive enzymes), colonize in the gut, and modulate the immune system [18]. Clinically, the DDS-1 strain has alleviated the symptoms associated with lactose intolerance in healthy volunteers between 18 to 75 years of age [24]. In addition, this particular probiotic strain has reduced symptoms of atopic dermatitis by improving immune markers and significantly shortened acute respiratory tract infection [25,26,27]. However, there are no studies on DDS-1 evaluating its capacity to alter the microbiota and metabolic phenotypes. The above-mentioned studies [18,24,27] encouraged us to hypothesize that the probiotic strain, *L. acidophilus* DDS-1, could further improve the beneficial microbial composition of healthy gut and host metabolism. Precise identification of metabolites associated with DDS-1 treatment throughout aging will be beneficial to identify disease-modifying, clinically accessible biomarkers for host health [28].

In the current study, we employed a healthy aging-based model to evaluate the direct effects of DDS-1 on gut microbiota changes associated with fecal metabolome composition. A combination of omics-based approaches, involving 16S rRNA gene sequencing and untargeted GC-MS based metabolomics, were utilized to provide a comprehensive understanding of changes in host metabolic phenotype of altering gut microbiota with and without DDS-1 supplementation.

## 2. Methods

### 2.1. Ethics Statement

All animal procedures were performed in accordance with the Australian Code of Practice for the Care and Use of Animals for Scientific Purposes of the National Health and Medical Research Council. The study was approved by the Animal Ethics Committee of the University of Tasmania (A0015840).

### 2.2. Bacterial Culture and Probiotic Feed Preparation

The bacterial strain utilized in the study, *L. acidophilus* DDS-1, was obtained in freeze-dried, free-flowing lyophilized form from UAS Labs, Madison, WI, USA. The bacterial culture was suspended in autoclaved water before making the probiotic-chow mix at a concentration of approximately 3 × 10^9^ CFU/g in a manner similar to Kuo et al. [29].

### 2.3. Animals and Probiotic Treatments

A total of 32 mice were used in the present study. Young (age = 3–4 weeks, *n* = 16) and aging (age = 35–36 weeks, *n* = 16) C57BL/6J mice of both sexes were obtained from the University of Tasmania animal breeding facility. Animals were housed within individually ventilated cages containing a corncob bedding (Andersons, Maumee, OH, USA), in a room with a temperature maintained at 21–22 °C, with a 12-h light/dark cycle. Mice were allowed access to radiation-sterilized rodent feed (Barastoc Rat and Mouse (102108), Ridley Agriproducts, Melbourne, Australia) and water available *ad libitum*. The young and aging mice were randomly divided into 4 groups (*n* = 8) and housed in individual cages containing (1) Young control group (YC) fed with normal chow, (2) Young probiotic group (YP) fed with probiotic chow, (3) Aging control group (AC) fed with normal chow, and (4) Aging probiotic group (AP) fed with probiotic chow. All the mice were fed for 4 weeks with group-specific chow

### 2.4. Fecal Sample Collection and Preparation

Throughout the experiment, the weight of each mouse was recorded each day and fecal samples were collected on the 28th day in a manner similar to Langille et al. [2]. To minimize contamination, on the day of sampling mice had no access to food and water, and sterile forceps were used for fecal sample collection. At least 2 pellets (100–150 mg of the sample) were collected from each mouse and immediately transferred into a sterile microcentrifuge tube, stored at −80 °C for 16S rRNA gene sequencing and metabolomic analysis.

### 2.5. Microbiota Analysis Using 16S rRNA High-Throughput Sequencing

The total DNA was extracted from fecal samples using the QIAamp DNA Stool Mini Kit (Qiagen, Melbourne, VIC, Australia). The samples underwent high-throughput sequencing on the Illumina MiSeq platform at the Australian Genome Research Facility (University of Queensland, Brisbane, QLD, Australia). PCR amplicons spanning the 16S rRNA V3-V4 hypervariable region with forward Primer (27F) AGAGTTTGATCMTGGCTCAG and reverse Primer (519R) GWATTACCGCGGCKGCTG were sequenced. Paired-end reads were assembled by aligning the forward and reverse reads using PEAR1 (version 0.9.5) [30]. Primers were identified and trimmed. Trimmed sequences were processed using Quantitative Insights into Microbial Ecology (QIIME 1.8) 4 USEARCH 2.3 (version 8.0.1623) and UPARSE software [31]. Using USEARCH tools, sequences were quality filtered, and full-length duplicate sequences were removed and sorted by abundance. Singletons or unique reads in the data set were discarded. Sequences were clustered followed by chimera filtered using “rdp_gold” database as a reference [32,33]. To obtain a number of reads in each Operational taxonomic units (OTUs), reads were mapped back to OTUs with a minimum identity of 97%. Using QIIME, taxonomy was assigned using Greengenes database5 (Version 13_8, August 2013). Image analysis was performed in real time by the MiSeq Control Software (MCS) v2.6.2.1 and Real-Time Analysis (RTA) v1.18.54, running on the instrument computer. RTA performs real-time base calling on the MiSeq instrument computer. Then the Illumina bcl2fastq 2.20.0.422 pipeline [32] was used to generate the sequence data. Next, 16S rRNA gene sequences were analyzed using MEGAN6 (Community edition version) [34], Microbiome analyst [35], and QIIME. Statistical analysis of Brady-Curtis dissimilarities calculated using the relative abundances of bacterial genera was conducted using Adonis function in R (version 3.2).

### 2.6. Fecal Metabolomics

The samples were subjected to derivatisation to increase volatility before subjecting to GC-MS analysis. Briefly, fecal samples (*n* = 5, weight = 40 mg) were freeze-dried and suspended in 1 mL methanol (LC-MS grade, Merck, Castle Hill, NSW, Australia), supplemented with 10 µg/mL adonitol (Analytical grade, Sigma Aldrich, Castle Hill, NSW, Australia) as an internal standard in a sterile 2 mL bead-beating tube. The samples were homogenized by bead beating for 30 s and then centrifuged at 570 g/4 °C for 15 min. The supernatant (50 µL) was transferred to a fresh centrifuge tube (1.5 mL) and dried in a vacuum evaporator centrifuge (LabGear, Brisbane, QLD, Australia) at 35 °C. Methoxyamine-HCl (20 mg/mL in Pyridine) (both, Analytical grade, Sigma Aldrich, Castle Hill, NSW, Australia) was added (40 µL) and samples were incubated at 30 °C/ 1400 rpm (ThermoMixer C, Eppendorf, Hamburg, Germany) for 90 min. This was followed by silylation with 70 µL BSTFA at 37 °C/1400 rpm for 30 min. Pre-derivatized 13C-Stearic acid (10 µg/mL) was added (1 µL) as the QA/QC internal standard. The mixture was briefly vortexed and centrifuged at 15,700 g for 5 min. The aliquot was transferred to vials for GC-MS analysis.

The GC-MS analysis was performed on an Agilent 6890B gas chromatograph (GC) oven coupled to a 5977B mass spectrometer (MS) detector (Agilent Technologies, Mulgrave, VIC, Australia) fitted with a MPS autosampler (Gerstel GmbH & Co. KG, Deutschland, Germany). The GC-MS conditions were as stated previously [36,37,38]. Data acquisition and spectral analysis were performed using the Qualitative Analysis software (Version B.08.00) of MassHunter workstation. Qualitative identification of the compounds was performed according to the Metabolomics Standard Initiative (MSI) chemical analysis workgroup [39] using standard GC-MS reference metabolite libraries (NIST 17, Fiehn Metabolomics RTL Library (G166766A, Agilent Technologies) and the Golm database) and with the use of Kovats retention indices based on a reference n-alkane standard (C8-C40 Alkanes Calibration Standard, Sigma-Aldrich, Castle Hill, NSW, Australia). For peak integration, a 5-point detection filtering (default settings) was set with a start threshold of 0.2 and a stop threshold of 0.0 for 10 scans per sample. Procedural blanks (*n* = 7) were analyzed randomly throughout the sequence batch. The obtained data was processed on Quantitative analysis platform of MassHunter workstation and exported as a Microsoft Excel output file for statistical analysis.

### 2.7. Data Analysis and Multivariate Analysis

GC-MS data imported to Microsoft Excel platform was normalized with respect to the internal standard adonitol (relative standard deviation = 11.257%). The normalized data was further log-transformed and auto-scaled (mean-centered) before statistical analysis [40]. To determine overall microbial variation in four groups, we used a principal coordinate analysis (PCoA), Unweighted Pair Group Method with Arithmetic Mean (UPGMA) dendrogram, Neighbour-Net (a distance-based method for constructing phylogenetic networks) hierarchical clustering with Brady-Curtis ecological indexing, Euclidean distances as the similarity measure, and Ward’s linkage as clustering algorithm [3,40]. For analysis of metabolome variations, Principal component analysis (PCA), partial least squares-discriminant analysis (PLS-DA) and orthogonal (O) PLS-DA were used. Because PLS-DA can overfit data, we used 1000 permutations to validate these models. The OPLS-DA was used to identify discrimination between metabolites contributing to classification. For heatmaps, the Euclidean distances were used as the similarity measure and Ward’s linkage as a clustering algorithm. Metabolite set enrichment analysis (MSEA) and Metabolic pathway analysis (MetPA) methods were used to perform correlation analysis and to identify treatment associated biochemical pathways [41].

### 2.8. Statistical Analysis

Graph Pad Prism version 7.0 for Windows was used for the statistical analysis. The data were analyzed using the Wilcoxon Mann–Whitney Test (multiple comparisons), with *p* < 0.05 set as the level of statistical significance. For microbial comparative analysis, a linear discriminant effect size (LEfSe) analysis was performed (α = 0.05), logarithmic Linear Discriminant Analysis (LDA) score threshold = 1.0. A MetaboAnalyst (Version 4.0) data annotation approach and Kyoto Encyclopaedia of Genes and Genomes (KEGG) Pathway Database were used for the hierarchical clustering analysis and significance analysis for microarrays (SAM), along with the variable importance of projection (VIP) [42]. The SAM and VIP methods are well-established statistical methods for metabolites and were used to select the most discriminant and interesting biomarkers [43].

## 3. Results

### 3.1. Body Weight During Days of Treatments

**Figure 1:**
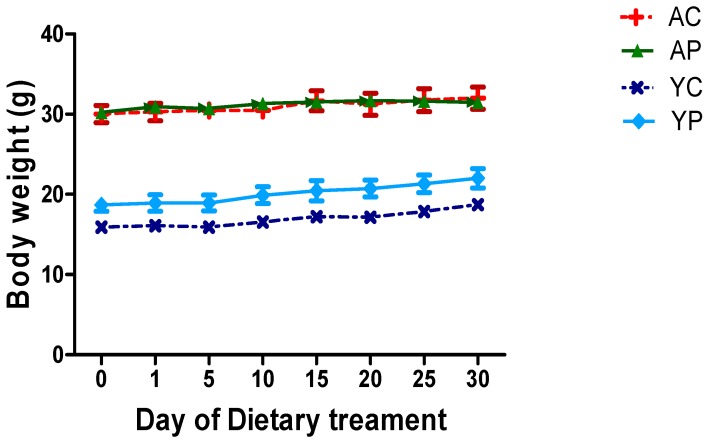
Effect of DDS-1 supplementation on body weights among the four groups. (YC) Young control group, (YP) young probiotic group, (AC) aging control group, (AP) aging probiotic group.

There was no significant change in body weights of mice treated with DDS-1 (Figure 1).

### 3.2. Gut Microbial Changes at the Phylum Level

**Figure 2:**
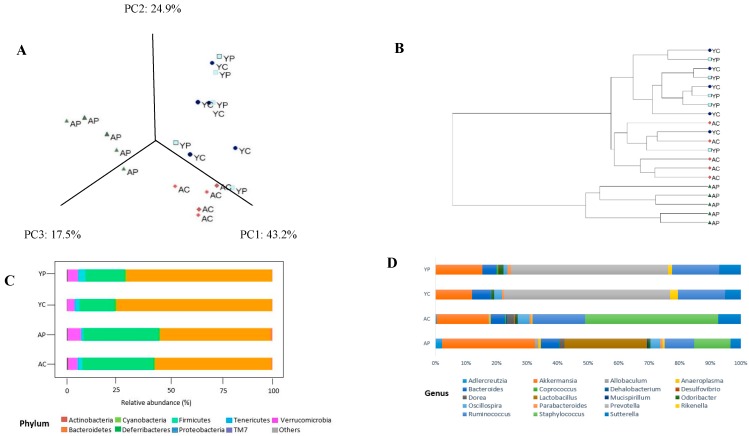
The gut microbiota changes observed in young control group (YC), young probiotic group (YP), aging control group (AC), aging probiotic group (AP) differentiated by principal coordinate analysis (PCoA) (**A**) and UPMGA dendrogram (**B**). The gut microbiota composition profiles at phylum (**C**) and genus levels (**D**) in control and probiotic-treated group, revealed by 16S rRNA gene sequencing (each color represents bacterial phylum and/or genus).

Fecal microbiota profiling was performed using 16S rRNA gene sequencing-based method. To compare the changes among the groups, PCoA was used. The PCoA plot of phylogeny with Brady-Curtis ecological indexing using ward clustering showed a clear separation of each group, with three distinct clusters (Figure 2A) at the OTU level among 4 groups. After DDS-1 treatment, both AP and YP groups segregated when compared to control groups. Particularly, the AP group more significantly separated from AC as depicted in UPGMA hierarchical clustering dendrogram (*p* < 0.01) (Figure 2B) and Neighbour-Net (Figure S1). Moreover, around 99% of the total microbial abundance was classified into nine major phyla in the aging group and seven in the young group, the rest were allocated as unclassified or others (Figure 2C). The dominant phyla were *Bacteroidetes* and *Firmicutes*, with their numbers higher in the young group (86.46 ± 2.10% and 8.20 ± 1.20%) compared with the aging group (63.35 ± 3.10% and 26.33 ± 4.45%) (*p* < 0.001).

**Figure 3:**
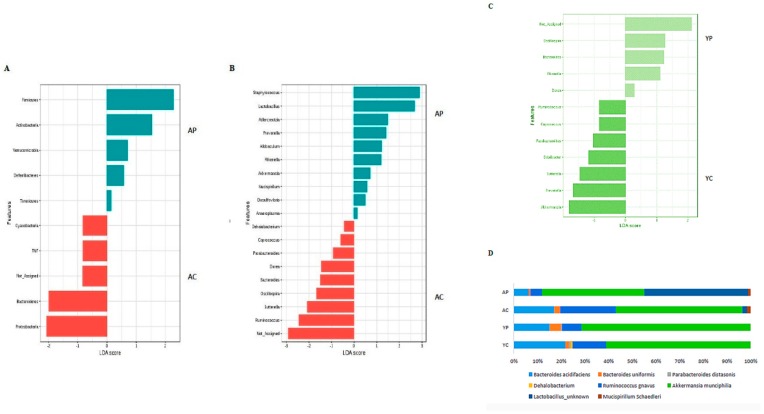
Comparative analysis with Linear Discriminant Analysis (LDA) Effect Size (LEfSe) scoring plot using Kruskal-Wallis rank sum test (*p* = 0.01 and log LDA threshold cut-off value = 1.0). Aging groups at Phylum (**A**) and genus (**B**) level. Young groups at genus (**C**) level. Significant changes at species (**D**) level. (YC) Young control group, (YP) young probiotic group, (AC) aging control group, (AP) aging probiotic group.

The treatment with DDS-1 helped increased *Firmicutes* levels from 8.40% to 9.32% (YP) (*p* < 0.001) and 26.33% to 30.72% (AP) (*p* < 0.001). However, it decreased the levels of *Bacteroidetes* in both the groups, reflecting the effect of DDS-1 supplementation on overall abundance of *Firmicutes. Verrucomicrobia* abundance was significantly increased in both treatment groups (YP: 0.72% to 2.10%; AP: 0.62 to 4.20%) (*p* < 0.001). *Proteobacteria* levels were decreased from 1.70% to 0.58% in the aging group after DDS-1 treatment. These changes in the aging groups were further confirmed with LEfSe analysis (α = 0.05), LDA approach keeping the cut-off value for *p* = 0.01 and log LDA threshold score = 1.0 (Figure 3A). In addition, under phylum *Bacteroidetes*, the relatively novel family *S24-7* were overrepresented in both the control groups (YC: 70.30% and AC: 52.8%) and their relative abundances were further decreased with DDS-1 treatment (YC: 67.50% and AC: 50.8%). Overall correlation analysis was performed using Spearman correlation analysis (Figure S2) with *p* < 0.01 as significant.

### 3.3. Gut Microbial Changes at Genus and Species Level Among Four Groups

At the genus level, the distribution of microbial populations of the aging groups was markedly different from the young groups. The AC group had significantly higher abundances of *Staphylococcus*, *Ruminococcus,* and *Sutterella* relative to the AP group. The DDS-1 treatment has increased the abundances of *Akkermansia*, *Adlercreutzia*, *Allobaculum*, *Rikenella,* and *Anaeroplasma*, while reduced the abundances of *Dorea*, *Oscillospira,* and *Ruminococcus* (Figure 2D). It is noteworthy that the *Lactobacillus* (*p* < 0.001) abundances significantly increase in AP group and was further confirmed with the LDA approach (Figure 3B). At the species level, DDS-1 treated groups have significantly increased *A. muciniphila* (*p* < 0.001) and decreased levels of *R*. *gnavus*, *Bacteroides acidifaciences*, and *B. uniformis* compared to the AC group (Figure 3D). Adding to this change, an abundance of *Mucispirillum schaedleri* (0.04 ± 0.02% to 0.10 ± 0.06%) was also increased in the AP group.

The YC group was enriched with *Prevotella*, *Ruminococcus,* and *Sutterella*, while very low levels of *Adlercreutzia*, *Allobaculum*, and *Alphaproteobacteria*, were identified compared to the AC group. *Anaeroplasma* and *Staphylococcus* were totally absent compared to the AC group. The treatment with DDS-1 increased the levels of *Akkermansia* (*p* < 0.05), *Parabacteroides*, and *Odoribacter* (Figure 2D). It also further increased *Prevotella* and *Sutterella* levels (*p* < 0.05) and was confirmed using the LDA approach (Figure 3C) in YP group. Similar to AP group, at the species level in YP group, *A. muciniphila* (*p* < 0.001) levels were considerably increased, and *R*. *gnavus*, *Bacteroides acidifaciences,* and *B. uniformis* levels were decreased compared to the YC group (Figure 3D). Certain bacterial species, such as *M. schaedleri,* were undetected in young groups. The beneficial effects of DDS-1 on modulating the gut microbiota were found to have a differential effect in between the two age groups. The treatment with DDS-1 significantly altered AP group, which was evident with the higher levels of *Lactobacillus* in fecal samples.

### 3.4. Metabolic Phenotyping of YC and YP Groups

**Figure 4:**
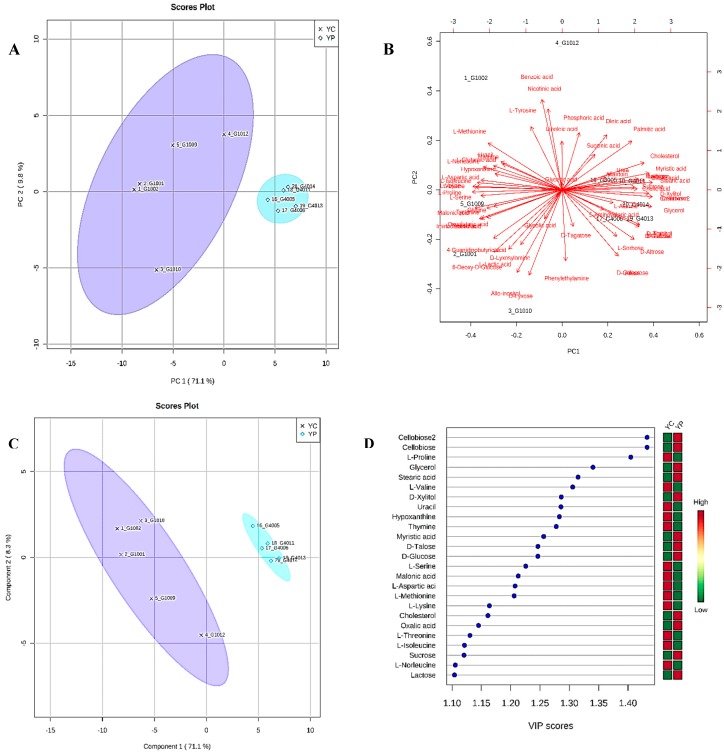
(**A**) Principal component analysis (PCA) plot of feces collected from young control (YC, violet color) and young treatment groups (YP, blue color) showing divergence. (**B**) Bi-plot showing compounds responsible for divergence. (**C**) 2D-PLS-DA plot showing spatial division among groups. (**D**) Key compounds separating YC and YP based on variable importance in projection (VIP) score plot in PLS-DA analysis.

To gain an untargeted overview of probiotic-induced changes in dominant gut metabolites, we analyzed fecal samples using a GC-MS platform. A total of 68 metabolites of different functional groups such as sugars, amino acids, SCFAs, and biogenic amines were detected. An unsupervised PCA was used to obtain an overview of the samples, and all samples were clearly discriminated, as shown in Figure 4A. PCA analysis showed a distinct clustering of the control group and DDS-1 treated group fecal samples. The biplot for PC1 and PC2 showed the compounds with the greatest impact on the division among the samples, as shown in Figure 4B. In order to provide a better visualization and to carry out the class separating information of each variable, a supervised PLS-DA approach was used to evaluate the metabolic patterns of YC group and YP group (Figure 4C). This analysis indicated the clear differences in the metabolic profiles of mice in YP and YC groups, which suggests that DDS-1 treatment induced significant biochemical changes. The accuracy, R^2^X, R^2^Y and Q^2^ of PLS-DA score analysis were 0.9, 0.86, and 0.82, respectively (Figure 4C), indicating the classification was well suited for the models, and the control and treated groups were classified clearly.

### 3.5. Identification of Potential Fecal Metabolites Associated with the YP/YC Groups

**Figure 5:**
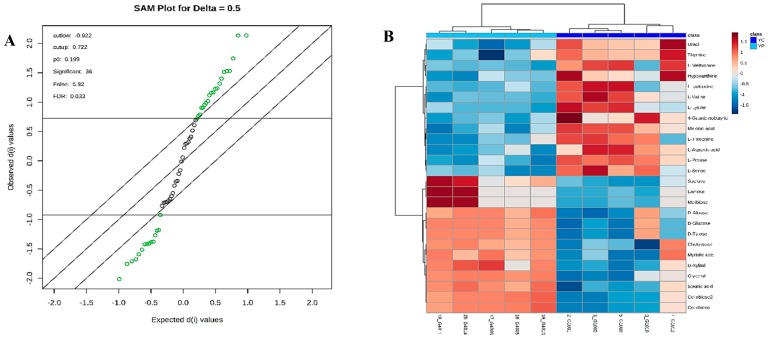
Significance analysis of microarray (SAM) plot (**A**) in positive mode (Delta score = 0.5, metabolites highlighted in green are significant). Significant changes in metabolites are expressed as a heat map (**B**) in the young group. (YC) Young Control group, (YP) young probiotic group.

The supervised *orthogonal* PLS-DA (OPLS-DA) was employed to further enhance the observed group separation in PCA and PLS-DA. Combination of PCA, PLS-DA, and VIP scores and O-PLS-DA (R^2^Y = 0.993 (*p* = 0.01), Q^2^ = 0.811 (*p* = 0.01)) (Figure S3A) and SAM enabled us to identify potential biomarkers (Figure 5A). The metabolites with VIP score > 1 and SAM (Figure 5A) could be considered as potential biomarkers responsible for altering the metabolic profile post DDS-1 treatment. The results showed 36 statistically significant metabolites contributing to the clustering, with their SAM scores and KEGG, InChI Key IDs listed in Table S1. Of the confirmed identifications, the most significant compounds were 4-Guanidinobutanoic acid (Fold changes (FC) = 149.21, *p* = 0.0055), Iminodiacetic acid (FC = 75.57, *p* = 0.022), L-Aspartic acid (FC = 47.02, *p* = 0.002), and L-Proline (FC = 31.96, *p* = 0.001). The varied tendencies of the identified fecal metabolites post DDS-1 treatment are depicted in the heat map (Figure 5B). Finally, the number of markers making a significant contribution was 21 in positive mode and 15 in negative mode using SAM and VIP analysis, as shown in Figure 4D.

### 3.6. Metabolic Phenotyping of AC and AP Groups

**Figure 6:**
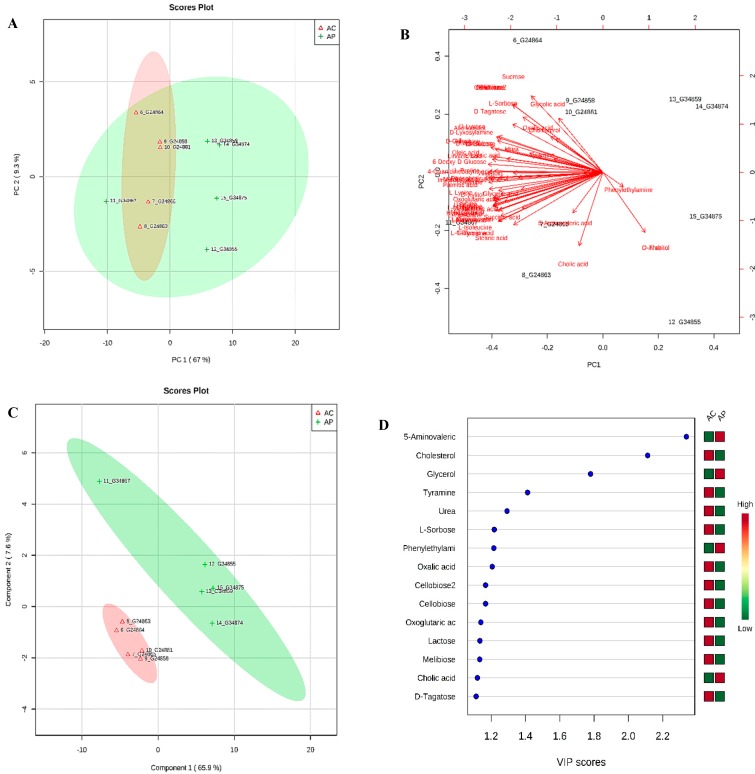
(**A**) PCA plot of feces collected from aging control (AC, red color) and aging probiotic groups (AP, green color) showing divergence. (**B**) Bi-plot showing compounds responsible for divergence (**C**) 2D-PLS-DA plot showing spatial division among groups (**D**) Key compounds separating AC and AP based on variable importance in projection (VIP) score plot in PLS-DA analysis.

Similar to young groups, the aging groups also yielded 68 metabolites of different functional groups such as sugars, amino acids, SCFAs, and biogenic amines. The PCA and PLS-DA models were performed to evaluate the metabolic phenotyping of the AC group and AP group (Figure 6A,C). The biplot for PC1 and PC2 showed the compounds with the greatest impact on the division among the samples, as shown in Figure 6B. The metabolic profile of mice in the AP group differed from the AC group, indicating significant biochemical changes induced by DDS-1. The accuracy, R^2^Y, and Q^2^ of PLS-DA score analysis were 0.763, 0.959, and 0.824, respectively (Figure 6C), indicating the classifications was well suited for the models, and the control and treated groups were classified clearly.

### 3.7. Identification of Potential Fecal Metabolites Associated with the AP/AC Groups

**Figure 7:**
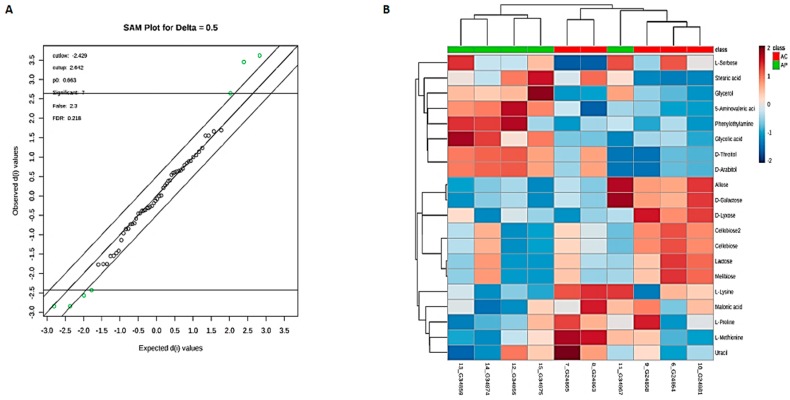
Significance analysis of microarray (SAM) plot (**A**) (Delta score = 0.5, metabolites highlighted in green are significant). Significant changes in metabolites are expressed as a heat map (**B**) in aging groups. (AC) Aging control group, (AP) Aging probiotic group.

The supervised OPLS-DA model was utilized to enhance the biomarker identification. The OPLS-DA method separated the control group and probiotic group into two different blocks, indicating better discrimination (R^2^Y= 0.994 (*p* = 0.01), Q^2^ = 0.863(*p* = 0.1)) (Figure S3B). From VIP scores (Figure 6D), PCA, PLS-DA, and O-PLS-DA approach, we could identify a total of 19 statistically significant metabolites that contribute to the overall cluster (Table S2). Out of identified 19 significantly changed metabolites in feces, 7 compounds were selected as potential biomarkers involved in the classification of AP gut using SAM analysis (Delta value = 0.5) (Figure 7A). Of the confirmed identifications, the most significant compounds were Lactose (Fold changes (FC) = 3.16, *p* = 0.003), Melibiose (FC = 3.13, *p* = 0.04), Cellobiose (FC = 3.06, *p* = 0.02). The varied tendencies of the identified fecal metabolites post DDS-1are depicted in the heat map for each group (Figure 7B).

### 3.8. Comparative Metabolite Phenotyping in Young and Aging Groups

**Figure 8:**
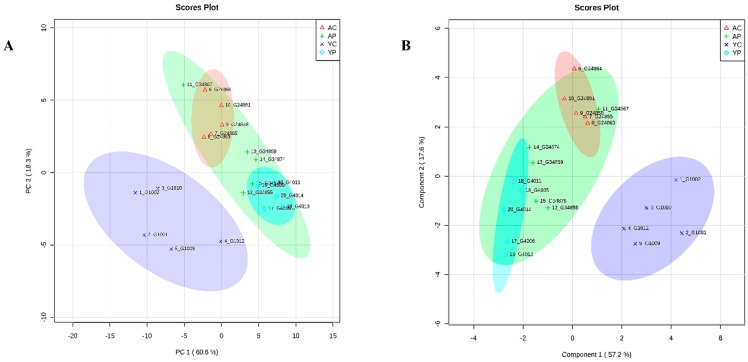
PCA plot (**A**) and PLS-DA plot (**B**) showing spatial division among all four groups. (YC) young control group, (YP) young probiotic group, (AC) aging control group, (AP) aging probiotic group.

PCA and PLS-DA analyses of the fecal metabolites showed a divergence between all four groups (Figure 8A,B). The samples from YC and AC were different from YP and AP groups, respectively (Figure S4A–D). Fecal samples from the groups YC and YP formed two clusters. Both of the clusters from the young groups were different and showed great divergence compared to the aging groups. Similarly, the AP group formed a cluster which was fairly different from the AC group and more closely related to the YP group (Figure 8B). The bi-plot for PC1 and PC2 shows the variation in the dataset and indicates that specific metabolites associated with the four groups are responsible for divergence among the samples (Figure S5A). VIP-value plot (Figure S5B) of the most significant metabolites and Figures S5–S8 presents heat map correlation analysis, showing the relative concentrations of significant metabolites in all four groups.

### 3.9. Identification of Key Metabolic Pathways Using MetPA

Detailed analysis of functional correlations and networks influenced throughout aging and with DDS-1 supplementation were performed by MetPA and MSEA methods. In order to identify the pathways that had an impact on altering gut microbiota-associated metabolic signature, the ions contributing to the separation of controls and probiotic-supplemented mice were analyzed using MetPA, MetaboAnalyst 4.0 (http://www.metaboanalyst.ca) [40,42]. A comprehensive metabolic network was mapped using joint pathway analysis by integration of all potential biomarkers identified in the current research.

**Figure 9:**
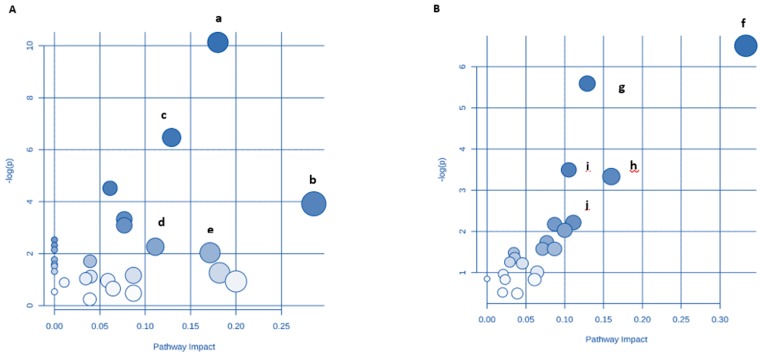
Potential pathways for the young probiotic group (YP) and aging probiotic group (AP), after DDS-1 supplementation, in fecal extracts identified using MetPA pathway analysis. YP group (**A**) with pathways (a) Valine, leucine, and isoleucine biosynthesis (b) Aminoacyl-tRNA biosynthesis (c) Glycine, serine, and threonine metabolism (d) Galactose metabolism (e) Cysteine and methionine metabolism. AP group (**B**) with pathways (f) D-Glutamine and D-glutamate metabolism (g) Alanine, aspartate, and glutamate metabolism (h) Galactose metabolism (i) Nitrogen metabolism (j) Butanoate metabolism.

In the young group, metabolic pathway analysis revealed that the metabolites which were identified as significant are important for host response to DDS-1 treatment (Figure 9A and Table S3). An impact value was set to 0.10 as a threshold, and any pathway receiving > 0.10 value was filtered out as potential target pathways [3,28,40,41,42]. These pathways include Valine, leucine, and isoleucine biosynthesis, Alanine, aspartate, and glutamate metabolism, Pentose and glucuronate interconversions, Aminoacyl-tRNA biosynthesis, Glycine, serine, and threonine metabolism, Galactose metabolism, and Cysteine and methionine metabolism. In the aging groups, a total of 5 metabolic pathways were identified (impact > 0.10) (Figure 9B and Table S4). D-glutamine and D-glutamate metabolism, alanine, aspartate and glutamate metabolism, galactose metabolism, nitrogen metabolism, and butanoate metabolism were recognized as key pathways in DDS-1-induced changes in metabolic phenotype of the aging gut. Omics Net was used to select and isolate individual KEGGS-genome-metabolite mapping (http://www.omicsnet.ca) [40,42] (Figures S9–S13). Of all the pathways identified, two pathways were found to be common among both age groups, specifically galactose metabolism and alanine, aspartate, and glutamate metabolism. These two pathways may play a role in the ability of LAB probiotics to modulate host metabolism via gut microbiota.

## 4. Discussion

In our study, we used a combination of 16S rRNA sequencing analysis and untargeted metabolomics profiling of fecal samples to identify specific gut microbiota changes and numerous gut metabolites that were associated with response to DDS-1 supplementation in young and aging mice. Importantly, DDS-1 increased *Firmicutes* abundance and enriched the populations of *Akkermansia* and *Lactobacillus*. Metabolic profiling of fecal samples indicated substantial changes to metabolites generated by gut microbes. Specifically, DDS-1 improved metabolism of pathways associated with amino acids, proteins, and carbohydrates.

Taxonomic differences were evaluated in this study among young and aging groups with and without DDS-1 treatment. In line with previous findings, our study confirms a microbial shift in the aging mouse gut [2,5,44]. At the phylum level, *Bacteroidetes* and *Firmicutes* were most dominant and their relative abundances were increased in the YC group compared to the AC group. *Acidobacteria* and *Tenericutes* were not detected in the young groups. DDS-1 treatment increased the *Firmicutes* population in both YP and AP groups, reflecting the role of *Lactobacillus* in overall abundance [2]. It is noteworthy that *Verrucomicrobia* levels were also significantly increased with DDS-1 treatment. Furthermore, *Proteobacteria* levels, which have been associated with inflammatory bowel disease (IBD) and colon cancer [4,16,45], were doubled in AC relative to the YC group. DDS-1 treatment was able to reduce their levels, indicating a potentially beneficial role as it relates to controlling intestinal inflammation [18,24,29]. Importantly, more than 50% of the mouse gut was enriched with a relatively new family *S-24-7*, that belongs to *Bacteroidetes* phylum. Only a few studies could hypothesize the role of *S-24-7* in butyrate production, however, the results were inconclusive and further research should be performed to understand its role [6,46].

We observed noticeable changes at the genus and species level within the aging mice groups. Interestingly, our data found an increase in abundance of *A. muciniphila* and *Lactobacillus* with DDS-1 treatment specific to the aging gut, which is consistent with previous studies [8,17,47]. Increase in relative abundance of the phylum *Firmicutes* could be associated with an increase of beneficial bacterial species, such as *Lactobacillus* species [16]. The mucin-feeding species, *A. muciniphila*, are thought to be biomarkers of intestinal health and their enrichment has been inversely correlated with IBD and metabolic disorders [2,16,44]. Recently, a study on aging obese mice demonstrated the extracellular vesicles of *A. muciniphila* improved body weight and lipid profiles of the mice concomitantly with metformin treatment [47]. In several prior studies, animals treated with *A. muciniphila* reported improvements in body weight, metabolic profiles, inflammation, and overall gut homeostasis by influencing immunity, mucin-layer thickness, and intestinal epithelial barrier proteins [1,44,47]. This was consistent with changes observed in body weights of the mice treated with DDS-1 in our study. Moreover, in our study, the levels of *Allobaculum* were increased in mice fed with DDS-1. Interestingly, the major end products of *Allobaculum* fermentation is butyrate (SCFA), which serves as a primary energy source in the gut [9]. In addition, bacterial species such as *R. gnavus* were present in both YC and AC groups. This particular member of *Clostridia* was directly correlated with the severity of IBD, suggesting its causal role in a dysbiotic shift in the aging gut [48]. DDS-1 supplementation was able to decrease *R. gnavus* abundance, and thus could possibly reduce inflammation associated with IBD or other conditions [18,24,26,27,48].

The small molecules present in feces are products of co-metabolism of microbes and host cells. Therefore, metabolite profiling in feces provides insight on novel metabolic biomarkers for health [3,28]. In an attempt to gain functional insight into changes in the gut microbiome, we have used a functional pathway analysis on probiotic-treated gut microbiota-derived metabolites. Untargeted metabolite profiling of control groups and DDS-1 supplemented groups not only allowed us to identify specific metabolites in the young and aging gut, but also to understand the mechanistic role of DDS-1. Our analysis revealed varying levels of metabolites in young and aging mice. The metabolite profile in the YC group was dissimilar from the AC group, which could be correlated with microbial shift [2,4,5]. Interestingly, we found that the metabolic phenotype of AP mice was clustered more towards YP mice (Figure 7B). The DDS-1 treatment allowed us to identify specific metabolites and their pathway-specific expressions profiles which demonstrated changes in metabolism are discussed below [41,49].

### 4.1. Amino Acid and Protein Metabolism

In many metabolic pathways, amino acids serve as crucial regulatory substrates [50]. Fecal branched-chain amino acids (BCAAs) such as valine, leucine, and isoleucine were significantly down-regulated in the YP group compared to the YC group. In particular, increases in a small cluster of essential amino acids including the BCAAs (i.e., leucine, valine, and isoleucine) are associated with a ~5-fold increased the risk of prospectively developing diabetes mellitus [3,6,41]. On the other hand, *Clostridium*, *Propionibacterium*, *Fusobacterium*, *Streptococcus*, and *Lactobacillus* bacteria are involved in the proteolytic activity to generate amino acids in the gut and linked to metabolic changes [49]. Specifically, altered levels of *Streptococcus* have been associated with metabolic disorders[6]. In our study, the relative abundance of *Streptococcus* is decreased in probiotic-treated groups. Moreover, glycine, serine, and threonine metabolism was increased in the YP group. This could be associated with decreased levels of L-serine in YP and AP, and these changes could be positively correlated with *Proteobacteria* and *Prevotella*, and negatively correlated with *Bacteroides* [3,51].

Glutamine, another nonessential amino acid and a precursor of glutamate, is able to promote beta-cells to secrete insulin under the stimulation of glucose. Glutamate, which can be converted into gamma-aminobutyric acid (GABA), is of vital importance in the decomposition of metabolites [52]. In addition, glutamate can also be converted into ornithine. On the other hand, aspartate can be transformed into asparagine and alanine. A study by Wang et al. [41] reported that elevated levels of alanine and aspartate have a positive association with obesity. In our study, the levels of alanine and aspartate were significantly decreased in the feces of the AP group, indicating a potentially beneficial role of DDS-1 in aging, which can be positively correlated with an increase in levels of *A. muciniphila*. In addition, the aging process can have a profound effect on protein metabolism, as well as overall nutritional status influenced by food intake [53,54]. Therefore, our results speculate that alanine, aspartate, and glutamate metabolism post-enrichment of *A. muciniphila* may potentially reduce the risk factors of metabolic syndrome.

Cysteine and methionine are the only two sulfur-containing amino acids that integrate into proteins [55]. Accumulating evidence indicates that the intestinal metabolism of dietary sulfur-containing amino acids methionine and cysteine are nutritionally important for normal intestinal mucosal growth [56]. The cysteine and methionine metabolism pathway was upregulated in YP group, which could be associated with beneficial modulation of gut microbiota and basic metabolism of growth in youth [55]. Our MetPA analysis indicated that L-serine, with importance score of 0.07407, was equally involved in the pathway. Studies have reported the role of the LAB, such as *Lactobacillus* species, in the biosynthesis of cysteine from serine, suggesting the role of DDS-1. In particular, this could be positively correlated with decreased levels of *Desulfovibrionales* in DDS-1 treated groups, which are associated with bacterial infections and Crohn’s disease [16].

Aminoacyl-tRNA (A-tRNA) plays a vital role in RNA translation (tRNA) and gene expression associated with protein biosynthesis [57]. Amino-acylation of tRNA involves A-tRNA synthetases to catalyze and form an association between cognate amino acid and tRNA, which is the initial step of protein synthesis. This involves in adenylation of amino acid to A-tRNA in adenosine triphosphate (ATP)-dependent manner to form Aminoacyl-adenosine monophosphate (AMP). This is followed by formation of A-tRNA and AMP in presence of A-AMP. In turn, an accurate A-tRNA biosynthesis is required for survival of all cells [58]. In addition, specific aminoacyl tRNA synthetases are implicated in various metabolic and neurological disorders [59]. Specifically, tetracyclines, a class of antibiotics, interfere with the initiation step of protein synthesis by inhibiting the binding of aminoacyl tRNA to the A-site of the ribosome, leading to bacteriostatic effect [60]. The activation of A-tRNA biosynthesis pathway could be an important factor in amino acid metabolism and activation of other amino acid pathways mentioned above [61]. It is noteworthy that A-tRNA biosynthesis pathway was only detected in the YP group. In particular, MetPA indicated that L-aspartic acid, L-serine, L-valine, L-lysine, L-threonine, and Isoleucine, with importance scores ≥ 0.02, were significantly involved in this pathway. The altered levels of the above-mentioned amino acids in YP were well correlated with the beneficial role of DDS-1 in protein synthesis and metabolism.

Nitrogen balance is an essential part of protein metabolism. The biological process of the nitrogen cycle is a complex interplay among many gut microbes catalyzing different reactions, where nitrogen is found in various oxidation states ranging from +5 in nitrate to −3 in ammonia [62]. Nitrogen metabolism pathway was only activated in the AP group when compared with the AC group. This could be linked to activation of amino acid metabolism, majorly, D-Glutamine and D-glutamate metabolism, where Glutamine (importance score = 0.11) can serve as a nitrogen donor in the production of amino sugars, nucleotides, and other amino acids [54,63]. Thus, glutamate/glutamine and aspartate are also associated with nitrogen metabolism (Figure S12B). On the other hand, removing amino acid nitrogen from the body is known as transaminations [64]. Transaminations are a class of reactions which helps in dissociation of nitrogen from amino acids either by deamination or oxidation process into a small group of compounds [65]. This result in ammonia production and their amine groups are converted to urea by the urea cycle [66]. Here, we used the total amount of urea present in the feces as a factor to measure nitrogen metabolism. Increased concentration of urea in the AP group indicates that DDS-1 has a regulatory effect on nitrogen metabolism perturbation [67].

### 4.2. Carbohydrate Metabolism

Digestion of lactose produces glucose and galactose, both of which are transported through the hepatic portal vein directly to the liver [54]. Galactose is metabolized by conversion initially to glucose 1-phosphate, which can then be converted either to glucose 6-phosphate or to glycogen. Galactose is crucial for human metabolism, with an established role in energy delivery and galactosylation of complex molecules [68]. Its main metabolic pathway is highly conserved in nature, being present in all living organisms. In humans, galactose is particularly important in early development. Genetic disorders that impair its metabolism inevitably cause disease, drawing attention to its key role [69]. Excess of galactose accumulation in tissue leads to various metabolic disorders. According to our MetPA analysis, metabolites such as D-galactose, melibiose, and lactose are shown to be directly involved in the D-galactose pathway. In our study, levels of these three metabolites were decreased in both YP and AP, which is directly correlated to active galactose metabolism demonstrating the beneficial role of DDS-1. Specifically, this could be correlated with increased in beneficial bacteria, such as *Akkermansia* species following probiotic treatment [3,17].

### 4.3. Butanoate Metabolism

The detailed functional analysis using MetPA analysis in our study indicated the importance of L-glutamic acid and oxoglutaric acid in Butanoate metabolism. Oxoglutaric levels were increased in AP when compared to AC. Butanoate or butyrate is a short chain fatty acid and secondary metabolite produced by gut microbes upon carbohydrate fermentation [7]. Although it was not detected in the untargeted metabolomics, butanoate (SCFA) could be linked to increased butyrate-producing bacteria in the gut. Bacteria classified under *Firmicutes* are directly involved in the Butanoate production [41]. In turn, SCFA act as a critical energy source for intestinal epithelial cells. Butanoate metabolism pathway activation in AP could be linked to increased relative abundances of *Firmicutes*, especially increased levels of *Lactobacillus*. *A. muciniphila* and *Allobaculum* enrichment in treatment groups is also positively correlated with SCFA production [16]. The DDS-1 treatment significantly ameliorated and modulated the gut microbiota associated metabolic pathways.

To the best of our knowledge, this is the first detailed study that demonstrates an integrated pattern recognition approach to understand the changes in the aging gut by exploring the metabolic network with and without probiotic supplementation. The key metabolic pathways identified by MetPA and MSEA play an important role in probiotic-induced gut microbiota-associated metabolic changes. Understanding the complex nature of specific gut microbiome and metabolome of the mouse, our study corroborates previous findings of gut microbiota shifts in aging could result in significant alterations in metabolic phenotype of the host with relevance to human health [2,5,47,54]. Any specific change to metabolic phenotype may lead to various GI and metabolic disorders [17,53,54]. The current model of probiotic-induced gut microbial and metabolic profiling is largely explained by a multi-omics approach using fecal 16S rRNA sequencing analysis and metabolomics [70]. Our results suggest that the aging-associated shifts in gut microbiota can be modulated by the dietary intervention of *L. acidophilus* DDS-1. In particular, this intervention enriched a number of beneficial bacteria, such as *Akkermansia*, *Allobaculum,* and *Lactobacillus* spp. in the gut, and directly contributed to the improvement of the host metabolic phenotype [8]. Translation of these outcomes to clinical accessibility warrants furthers larger mouse cohorts with extreme older mice before clinical studies. Indeed, DDS-1 intervention intended to selectively promote bacteria could become a vital dietary strategy to counteract aging-associated dysbiosis.

## 5. Conclusions

Our results highlight the key role of beneficial microbes/probiotics in determining and modulating the metabolic profile in the healthy-aging gut. Similar approaches could be used as a reference to identify and target specific health-promoting metabolic pathways in young and aging populations.

## Supplementary Materials

The following are available online at http://www.mdpi.com/2072-6643/10/9/1255/s1, Figure S1: Neighbour-net showcasing the three different clusters of all the samples using Brady-Curtis Ecological Index and Ward clustering (p < 0.01). (YC) Young control group, (YP) young probiotic group, (AC) aging control group, (AP) aging probiotic group, Figure S2: Heat map showing the Correlation analysis of fecal microbiota in aging and young groups at phylum level using Pearson r distance method. (*p* < 0.01), Figure S3: 2D O-PLS-DA plot with showing clear separation between YC and YP groups. (YC) Young control group, (YP) young probiotic group, (AC) aging control group, (AP) aging probiotic group, Figure S4: PCA plot of feces collected from YC and AC (A) and YP and AP (C) groups showing divergence. Key compounds separating YC and AC groups (B) based on variable importance in projection (VIP) score plot (D) in PLS-DA analysis. (YC) Young control group, (YP) young probiotic group, (AC) aging control group, (AP) aging probiotic group, Figure S5: A. Bi-plot showing compounds responsible for divergence in young and aging groups B. Key compounds separating young and aging groups based on variable importance in projection (VIP) score plot in PLS-DA analysis, Figure S6: Significant changes in metabolites are expressed as heat map in all four groups. (YC) Young control group, (YP) young probiotic group, (AC) aging control group, (AP) aging probiotic group, Figure S7: Overall metabolite cluster in YC, YP, AC and AP groups using Pearson’s R correlation with ward distancing method (*p* < 0.01). (YC) Young control group, (YP) young probiotic group, (AC) aging control group, (AP) aging probiotic group, Figure S8: Age-wise and Group-wise correlation analysis showing the cluster of YP and AP and differences in YC and AC groups using Pearson’s correlation using ward distancing method (*p* < 0.01). (YC) Young control group, (YP) young probiotic group, (AC) aging control group, (AP) aging probiotic group, Figure S9: Potential pathways for Young probiotic (YP) from Figure 8A (A) Valine, leucine and isoleucine biosynthesis, (B) Glycine, serine and threonine metabolism, (C) Aminoacyl-tRNA biosynthesis. Compounds in red are significantly involved in the pathways, Figure S10: Potential pathways for Aging probiotic (AP) from Figure 8B, (A) D-Glutamine and D-glutamate metabolism (B) Alanine, aspartate and glutamate metabolism (C) Nitrogen metabolism, (D) Butanoate metabolism. Compounds in red are significantly involved in the pathways, Figure S11: KEGGS pathways of Alanine, aspartate and glutamate metabolism (A) and Galactose Metabolism (B). Compounds highlighted in red and green are directly involved in respective pathways, Figure S12: KEGGS pathways of Butanoate metabolism (A) and Nitrogen Metabolism (B). Compounds highlighted in red box and green are directly involved in respective pathways, Table S1: Most significant compounds identified in YP/YC groups, Table S2: Most significant compounds identified by OPLS-DA and SAM analysis in AP group. (*First 7 compounds are identified by SAM).

## References

[1] Vemuri R., Gundamaraju R., Shastri M.D., Shukla S.D., Kalpurath K., Ball M., Tristram S., Shankar E.M., Ahuja K., Eri R. (2018). Gut microbial changes, interactions, and their implications on human lifecycle: An ageing perspective. BioMed Res. Int..

[2] Langille M.G., Meehan C.J., Koenig J.E., Dhanani A.S., Rose R.A., Howlett S.E., Beiko R.G. (2014). Microbial shifts in the aging mouse gut. Microbiome.

[3] Yu M., Jia H.-M., Zhou C., Yang Y., Sun L.-L., Zou Z.-M. (2017). Urinary and fecal metabonomics study of the protective effect of chaihu-shu-gan-san on antibiotic-induced gut microbiota dysbiosis in rats. Sci. Rep..

[4] Biagi E., Nylund L., Candela M., Ostan R., Bucci L., Pini E., Nikkïla J., Monti D., Satokari R., Franceschi C. (2010). Through ageing, and beyond: Gut microbiota and inflammatory status in seniors and centenarians. PLoS ONE.

[5] Fahlström A., Yu Q., Ulfhake B. (2011). Behavioral changes in aging female c57bl/6 mice. Neurobiol. Aging.

[6] Krych Ł., Nielsen D.S., Hansen A.K., Hansen C.H.F. (2015). Gut microbial markers are associated with diabetes onset, regulatory imbalance, and ifn-γ level in nod mice. Gut Microbes.

[7] Vemuri R., Gundamaraju R., Eri R. (2017). Role of lactic acid probiotic bacteria in ibd. Curr. Pharm. Des..

[8] Schneeberger M., Everard A., Gómez-Valadés A.G., Matamoros S., Ramírez S., Delzenne N.M., Gomis R., Claret M., Cani P.D. (2015). Akkermansia muciniphila inversely correlates with the onset of inflammation, altered adipose tissue metabolism and metabolic disorders during obesity in mice. Sci. Rep..

[9] Sybille T., June Z., Michael K., Roy M., Maria L.M. (2013). The intestinal microbiota in aged mice is modulated by dietary resistant starch and correlated with improvements in host responses. FEMS Microbiol. Ecol..

[10] Azzu V., Valencak T.G. (2017). Energy metabolism and ageing in the mouse: A mini-review. Gerontology.

[11] Valdes A.M., Glass D., Spector T.D. (2013). Omics technologies and the study of human ageing. Nat. Rev. Genet..

[12] Rampelli S., Candela M., Turroni S., Biagi E., Collino S., Franceschi C., O’Toole P.W., Brigidi P. (2013). Functional metagenomic profiling of intestinal microbiome in extreme ageing. Aging (Albany N. Y.).

[13] Son N., Hur H.J., Sung M.J., Kim M.-S., Hwang J.-T., Park J.H., Yang H.J., Kwon D.Y., Yoon S.H., Chung H.Y. (2012). Liquid chromatography–mass spectrometry-based metabolomic analysis of livers from aged rats. J. Proteome Res..

[14] Deda O., Gika H.G., Taitzoglou I., Raikos Ν., Theodoridis G. (2017). Impact of exercise and aging on rat urine and blood metabolome. An lc-ms based metabolomics longitudinal study. Metabolites.

[15] Kim S., Cheon H.-S., Song J.-C., Yun S.-M., Park S.I., Jeon J.-P. (2014). Aging-related changes in mouse serum glycerophospholipid profiles. Osong Public Health Res. Perspect..

[16] Shi Y., Zhao X., Zhao J., Zhang H., Zhai Q., Narbad A., Chen W. (2018). A mixture of lactobacillus species isolated from traditional fermented foods promote recovery from antibiotic-induced intestinal disruption in mice. J. Appl. Microbiol..

[17] Vemuri R., Gundamaraju R., Shinde T., Eri R. (2017). Therapeutic interventions for gut dysbiosis and related disorders in the elderly: Antibiotics, probiotics or faecal microbiota transplantation?. Benef. Microbes.

[18] Vemuri R., Shinde T., Shastri M.D., Perera A.P., Tristram S., Martoni C.J., Gundamaraju R., Ahuja K.D., Ball M., Eri R. (2018). A human origin strain lactobacillus acidophilus dds-1 exhibits superior in vitro probiotic efficacy in comparison to plant or dairy origin probiotics. Int. J. Med. Sci..

[19] Nagata S., Asahara T., Wang C., Suyama Y., Chonan O., Takano K., Daibou M., Takahashi T., Nomoto K., Yamashiro Y. (2016). The effectiveness of lactobacillus beverages in controlling infections among the residents of an aged care facility: A randomized placebo-controlled double-blind trial. Ann. Nutr. Metab..

[20] Van den Nieuwboer M., Klomp-Hogeterp A., Verdoorn S., Metsemakers-Brameijer L., Vriend T., Claassen E., Larsen O. (2015). Improving the bowel habits of elderly residents in a nursing home using probiotic fermented milk. Benef. Microbes.

[21] Rampelli S., Candela M., Severgnini M., Biagi E., Turroni S., Roselli M., Carnevali P., Donini L., Brigidi P. (2013). A probiotics-containing biscuit modulates the intestinal microbiota in the elderly. J. Nutr. Health Aging.

[22] Pellino G., Sciaudone G., Candilio G., Camerlingo A., Marcellinaro R., De Fatico S., Rocco F., Canonico S., Riegler G., Selvaggi F. (2013). Early postoperative administration of probiotics versus placebo in elderly patients undergoing elective colorectal surgery: A double-blind randomized controlled trial. BMC Surg..

[23] Allen S., Wareham K., Wang D., Bradley C., Sewell B., Hutchings H., Harris W., Dhar A., Brown H., Foden A. (2013). Pwe-008 placide: Probiotics in the prevention of antibiotic associated diarrhoea (aad) and clostridium difficile associated diarrhoea (cdd) in elderly patients admitted to hospital–results of a large multi-centre rct in the uk. Gut.

[24] Pakdaman M.N., Udani J.K., Molina J.P., Shahani M. (2015). The effects of the dds-1 strain of lactobacillus on symptomatic relief for lactose intolerance-a randomized, double-blind, placebo-controlled, crossover clinical trial. Nutr. J..

[25] Hulshof L., van’t Land B., Sprikkelman A.B., Garssen J. (2017). Role of microbial modulation in management of atopic dermatitis in children. Nutrients.

[26] Gerasimov S.V., Vasjuta V.V., Myhovych O.O., Bondarchuk L.I. (2010). Probiotic supplement reduces atopic dermatitis in preschool children. Am. J. Clin. Dermatol..

[27] Gerasimov S., Ivantsiv V., Bobryk L., Tsitsura O., Dedyshin L., Guta N., Yandyo B. (2016). Role of short-term use of l. Acidophilus dds-1 and b. Lactis uabla-12 in acute respiratory infections in children: A randomized controlled trial. Eur. J. Clin. Nutr..

[28] Wang X., Yang B., Zhang A., Sun H., Yan G. (2012). Potential drug targets on insomnia and intervention effects of jujuboside a through metabolic pathway analysis as revealed by uplc/esi-synapt-hdms coupled with pattern recognition approach. J. Proteom..

[29] Kuo S.-M., Merhige P.M., Hagey L.R. (2013). The effect of dietary prebiotics and probiotics on body weight, large intestine indices, and fecal bile acid profile in wild type and il10−/−mice. PLoS ONE.

[30] Zhang J., Kobert K., Flouri T., Stamatakis A. (2013). Pear: A fast and accurate illumina paired-end read merger. Bioinformatics.

[31] Edgar R.C. (2013). Uparse: Highly accurate otu sequences from microbial amplicon reads. Nat. Methods.

[32] Kuczynski J., Stombaugh J., Walters W.A., González A., Caporaso J.G., Knight R. (2012). Using qiime to analyze 16s rrna gene sequences from microbial communities. Curr. Protoc. Microbiol..

[33] Edgar R.C., Haas B.J., Clemente J.C., Quince C., Knight R. (2011). Uchime improves sensitivity and speed of chimera detection. Bioinformatics.

[34] Huson D.H., Mitra S. (2012). Introduction to the analysis of environmental sequences: Metagenomics with megan. Evolutionary Genomics.

[35] Dhariwal A., Chong J., Habib S., King I.L., Agellon L.B., Xia J. (2017). Microbiomeanalyst: A web-based tool for comprehensive statistical, visual and meta-analysis of microbiome data. Nucleic Acids Res..

[36] Beale D.J., Marney D., Marlow D.R., Morrison P.D., Dunn M.S., Key C., Palombo E.A. (2013). Metabolomic analysis of cryptosporidium parvum oocysts in water: A proof of concept demonstration. Environ. Pollut..

[37] Karpe A.V., Beale D.J., Harding I.H., Palombo E.A. (2015). Optimization of degradation of winery-derived biomass waste by ascomycetes. J. Chem. Technol. Biotechnol..

[38] Beale D., Morrison P., Key C., Palombo E. (2014). Metabolic profiling of biofilm bacteria known to cause microbial influenced corrosion. Water Sci. Technol..

[39] Sansone S.A., Fan T., Goodacre R., Griffin J.L., Hardy N.W., Kaddurah-Daouk R., Kristal B.S., Lindon J., Mendes P., Morrison N. (2007). The metabolomics standards initiative. Nat. Biotechnol..

[40] French K.E., Harvey J., McCullagh J.S. (2018). Targeted and untargeted metabolic profiling of wild grassland plants identifies antibiotic and anthelmintic compounds targeting pathogen physiology, metabolism and reproduction. Sci. Rep..

[41] Wang M., Zhang X., Wang Y., Li Y., Chen Y., Zheng H., Ma F., Ma C.W., Lu B., Xie Z. (2018). Metabonomic strategy for the detection of metabolic effects of probiotics combined with prebiotic supplementation in weaned rats. RSC Adv..

[42] Sun H., Zhang A., Yan G., Piao C., Li W., Sun C., Wu X., Li X., Chen Y., Wang X. (2012). Metabolomic analysis of key regulatory metabolites in hcv-infected tree shrews. Mol. Cell. Proteom..

[43] Hayakawa K., Matsuda F., Shimizu H. (2016). Metabolome analysis of saccharomyces cerevisiae and optimization of culture medium for s-adenosyl-l-methionine production. AMB Express.

[44] Cani P.D. (2018). Human gut microbiome: Hopes, threats and promises. Gut.

[45] Robinson A.M., Gondalia S.V., Karpe A.V., Eri R., Beale D.J., Morrison P.D., Palombo E.A., Nurgali K. (2016). Fecal microbiota and metabolome in a mouse model of spontaneous chronic colitis: Relevance to human inflammatory bowel disease. Inflamm. Bowel Dis..

[46] Welly R.J., Liu T.-W., Zidon T.M., Rowles III J.L., Park Y.-M., Smith T.N., Swanson K.S., Padilla J., Vieira-Potter V.J. (2016). Comparison of diet vs. Exercise on metabolic function & gut microbiota in obese rats. Med. Sci. Sports Exerc..

[47] Lee H., Lee Y., Kim J., An J., Lee S., Kong H., Song Y., Lee C.-K., Kim K. (2018). Modulation of the gut microbiota by metformin improves metabolic profiles in aged obese mice. Gut Microbes.

[48] Hall A.B., Yassour M., Sauk J., Garner A., Jiang X., Arthur T., Lagoudas G.K., Vatanen T., Fornelos N., Wilson R. (2017). A novel ruminococcus gnavus clade enriched in inflammatory bowel disease patients. Genome Med..

[49] Vital M., Howe A.C., Tiedje J.M. (2014). Revealing the bacterial butyrate synthesis pathways by analyzing (meta) genomic data. MBio.

[50] Neis E.P., Dejong C.H., Rensen S.S. (2015). The role of microbial amino acid metabolism in host metabolism. Nutrients.

[51] Vernocchi P., Del Chierico F., Quagliariello A., Ercolini D., Lucidi V., Putignani L. (2017). A metagenomic and in silico functional prediction of gut microbiota profiles may concur in discovering new cystic fibrosis patient-targeted probiotics. Nutrients.

[52] Hertz L. (2013). The glutamate–glutamine (gaba) cycle: Importance of late postnatal development and potential reciprocal interactions between biosynthesis and degradation. Front. Endocrinol. (Lausanne).

[53] Fisher D.Y., Fukagawa N.K. (2017). Protein and amino acid metabolism in the elderly. Methods for Investigation of Amino Acid and Protein Metabolism.

[54] Munro H.N., Gersovitz M., Young V.R. (2018). Human aging: Protein and amino acid metabolism and implications for protein and amino acid requirements. Nutritional Approaches to Aging Research.

[55] Bauchart-Thevret C., Stoll B., Burrin D.G. (2009). Intestinal metabolism of sulfur amino acids. Nutr. Res. Rev..

[56] Bogicevic B., Berthoud H., Portmann R., Bavan T., Meile L., Irmler S. (2016). Cysteine biosynthesis in lactobacillus casei: Identification and characterization of a serine acetyltransferase. FEMS Microbiol. Lett..

[57] Hausmann C.D., Ibba M. (2008). Aminoacyl-trna synthetase complexes: Molecular multitasking revealed. FEMS Microbiol. Rev..

[58] Bhatt T.K., Kapil C., Khan S., Jairajpuri M.A., Sharma V., Santoni D., Silvestrini F., Pizzi E., Sharma A. (2009). A genomic glimpse of aminoacyl-trna synthetases in malaria parasite plasmodium falciparum. BMC Genomics.

[59] Park S.G., Schimmel P., Kim S. (2008). Aminoacyl trna synthetases and their connections to disease. Proc. Natl. Acad. Sci. USA.

[60] Jafarnejad S.M., Kim S.-H., Sonenberg N. (2018). Aminoacylation of proteins: New targets for the old arsenal. Cell Metab..

[61] Kong J., Fang P., Madoux F., Spicer T.P., Scampavia L., Kim S., Guo M. (2018). High-throughput screening for protein synthesis inhibitors targeting aminoacyl-trna synthetases. SLAS Discov..

[62] Finkel T. (2015). The metabolic regulation of aging. Nat. Med..

[63] De Las Heras J., Aldámiz-Echevarría L., Martínez-Chantar M.-L., Delgado T.C. (2017). An update on the use of benzoate, phenylacetate and phenylbutyrate ammonia scavengers for interrogating and modifying liver nitrogen metabolism and its implications in urea cycle disorders and liver disease. Expert Opin. Drug Metab. Toxicol..

[64] Yudkoff M. (2017). Interactions in the metabolism of glutamate and the branched-chain amino acids and ketoacids in the cns. Neurochem. Res..

[65] Garibotto G., Verzola D., Vettore M., Tessari P. (2017). The contribution of muscle, kidney, and splanchnic tissues to leucine transamination in humans. Can. J Physiol. Pharmacol..

[66] Waisbren S.E., Cuthbertson D., Burgard P., Holbert A., McCarter R., Cederbaum S., Consortium U.C.D. (2018). Biochemical markers and neuropsychological functioning in distal urea cycle disorders. Pharmacol. Res. J Inherit. Metab. Dis..

[67] Davila-Gay A.-M., Blachier F., Gotteland M., Andriamihaja M., Benetti P.-H., Sanz Y., Tomé D. (2013). Intestinal luminal nitrogen metabolism: Role of the gut microbiota and consequences for the host. Pharmacol. Res..

[68] Coelho A.I., Berry G.T., Rubio-Gozalbo M.E. (2015). Galactose metabolism and health. Curr. Opin. Clin. Nutr. Metab. Care.

[69] Hobbs M.E., Williams H.J., Hillerich B., Almo S.C., Raushel F.M. (2014). L-galactose metabolism in bacteroides vulgatus from the human gut microbiota. Biochemistry.

[70] Gundamaraju R., Vemuri R., Eri R., M Ishiki H., Coy-Barrera E., Sastry Yarla N., Golzio dos Santos S., Feitosa Alves M., Barbosa Filho M., FFM Diniz M. (2017). Metabolomics as a functional tool in screening gastro intestinal diseases: Where are we in high throughput screening?. Comb. Chem. High Throughput Screen..

