# Supplementary material for: Lactobacillus acidophilus DDS-1 Modulates the Gut Microbiota and Improves Metabolic Profiles in Aging Mice
Source: Nutrients. 2018 Sep 6;10(9):1255. doi: 10.3390/nu10091255 (PMC6165029; doi:10.3390/nu10091255)
Supplement: Supplementary file 1 [file nutrients-10-01255-s001.zip › Supplementary Tables.docx]

**Table S1: Most significant compounds identified in YP/YC groups**

| **Compound Name** | **InChI Key** | **KEGG** | **FC** | **Log2(FC)** | **SAM (*p* value)** |
| --- | --- | --- | --- | --- | --- |
| Cellobiose | GUBGYTABKSRVRQQRZGKKJRA-N | C06422 | 0.030 | -5.010 | 0.00033333 |
| Cellobiose2 | GUBGYTABKSRVRQQRZGKKJRA-N | - | 0.030 | -5.019 | 0.00066667 |
| L-Proline | ONIBWKKTOPOVIA-BYPYZUCNSA-N | C00148 | 31.966 | 4.998 | 0.001 |
| L-Aspartic acid | CKLJMWTZIZZHCS-REOHCLBHSA-N | C00049 | 47.016 | 5.555 | 0.002 |
| Glycerol | PEDCQBHIVMGVHV-UHFFFAOYSA-N | C00116 | 0.079 | -3.650 | 0.0023333 |
| Malonic acid | OFOBLEOULBTSOW-UHFFFAOYSA-N | C00383 | 22.051 | 4.462 | 0.0028333 |
| L-Serine | MTCFGRXMJLQNBG-REOHCLBHSA-N | C00065 | 34.464 | 5.107 | 0.0031667 |
| L-Valine | KZSNJWFQEVHDMF-BYPYZUCNSA-N | C00183 | 28.623 | 4.839 | 0.0035 |
| Sucrose | CZMRCDWAGMRECN-UGDNZRGBSA-N | C00089 | 0.024 | -5.363 | 0.0046667 |
| D-Glucose | WQZGKKKJIJFFOK-GASJEMHNSA-N | C00031 | 0.164 | -2.599 | 0.005 |
| 4-Guanidinobutanoic acid | TUHVEAJXIMEOSA-UHFFFAOYSA-N | C01035 | 149.21 | 7.221 | 0.0055 |
| GDP-6-deoxy-D-talose | LQEBEXMHBLQMDB-UUZHTGJLSA-N | C02977 | 0.180 | -2.473 | 0.0058333 |
| Thymine | RWQNBRDOKXIBIV-UHFFFAOYSA-N | C00178 | 11.418 | 3.513 | 0.0088333 |
| Uracil | ISAKRJDGNUQOIC-UHFFFAOYSA-N | C00106 | 9.485 | 3.245 | 0.0091667 |
| L-Methionine | FFEARJCKVFRZRR-BYPYZUCNSA-N | C00073 | 22.344 | 4.481 | 0.0105 |
| Stearic acid | QIQXTHQIDYTFRH-UHFFFAOYSA-N | C01530 | 0.222 | -2.169 | 0.011167 |
| L-Lysine | KDXKERNSBIXSRK-YFKPBYRVSA-N | C00047 | 26.333 | 4.718 | 0.012167 |
| L-Threonine | AYFVYJQAPQTCCC-GBXIJSLDSA-N | C00188 | 25.073 | 4.648 | 0.0125 |
| Hypoxanthine | FDGQSTZJBFJUBT-UHFFFAOYSA-N | C00262 | 12.996 | 3.701 | 0.016333 |
| Alpha-Lactose | GUBGYTABKSRVRQ-QKKXKWKRSA-N | C00243 | 0.029 | 3.245 | 0.0185 |
| Melibiose | DLRVVLDZNNYCBX-ABXHMFFYSA-N | C05402 | 0.029 | -5.079 | 0.019333 |
| Iminodiacetic acid | NBZBKCUXIYYUSX-UHFFFAOYSA-N | C19911 | 75.569 | 6.239 | 0.022 |
| L-Isoleucine | AGPKZVBTJJNPAG-WHFBIAKZSA-N | C00407 | 20.298 | 4.343 | 0.023167 |
| Cholesterol | HVYWMOMLDIMFJA-DPAQBDIFSA-N | C00187 | 0.344 | -1.538 | 0.026167 |
| D-Xylitol | HEBKCHPVOIAQTA-NGQZWQHPSA-N | C00379 | 0.340 | -1.555 | 0.026667 |
| Urea | XSQUKJJJFZCRTK-UHFFFAOYSA-N | C00086 | 0.005 | -7.569 | 0.031667 |
| Allose | WQZGKKKJIJFFOK-IVMDWMLBSA-N | C01487 | 0.257 | - | 0.043667 |
| D-Galactose | WQZGKKKJIJFFOK-PHYPRBDBSA-N | C00984 | 4.167 | 2.059 | 0.049 |
| Myristic acid | TUNFSRHWOTWDNC-UHFFFAOYSA-N | C06424 | 0.445 | -1.165 | 0.053667 |
| L-Norleucine | LRQKBLKVPFOOQJ-YFKPBYRVSA-N | C01933 | 13.767 | 3.783 | 0.059667 |
| D-Arabitol | HEBKCHPVOIAQTA-QWWZWVQMSA-N | C01904 | 0.431 | -1.221 | 0.064333 |
| D-Threitol | UNXHWFMMPAWVPIQWWZWVQMA-N | C16884 | 0.431 | -1.211 | 0.064333 |
| L-Sorbose | LKDRXBCSQODPBY-AMVSKUEXSA-N | C00247 | 0.28365 | -1.817 | 0.111 |
| Oxalic acid | MUBZPKHOEPUJKR-UHFFFAOYSA-N | C00209 | 0.44955 | -1.153 | 0.124 |
| 5-Aminopentanoic acid | JJMDCOVWQOJGCB-UHFFFAOYSA-N | C00431 | 0.44995 | - | 0.143 |

**(**International Chemical Identifiers (InChI) and standard InChI hashes (InChIKey); KEGG = Kyoto Encyclopaedia of Genes and Genomes;

FC= fold change)

**Table S2**. Most significant compounds identified by OPLS-DA and SAM analysis in AP group. (*****First 7 compounds are identified by SAM)

| **Compound Name** | **InChI Key** | **KEGG** | **FC** | **Log2(FC)** | **SAM (p values)** |
| --- | --- | --- | --- | --- | --- |
| Glycerol* | PEDCQBHIVMGVHV-UHFFFAOYSA-N | C00116 | 0.252 | -1.985 | 0.01 |
| 5-Aminopentanoic acid* | JJMDCOVWQOJGCB-UHFFFAOYSA-N | C00431 | 0.219 | -2.190 | 0.0115 |
| Cellobiose* | GUBGYTABKSRVRQ-QRZGKKJRSA-N | C06422 | 3.068 | 1.617 | 0.0255 |
| Cellobiose2* | GUBGYTABKSRVRQ-QRZGKKJRSA-N | C06422 | 3.066 | 1.618 | 0.0255 |
| Phenyl ethylamine* | BHHGXPLMPWCGHP-UHFFFAOYSA-N | C05332 | 0.052 | -4.257 | 0.032833 |
| Lactose* | GUBGYTABKSRVRQQKKXKWKRSA-N | C00243 | 3.163 | 1.661 | 0.0365 |
| Melibiose* | DLRVVLDZNNYCBX-ABXHMFFYSA-N | C05402 | 3.131 | 1.646 | 0.043 |
| Cholic acid | BHQCQFFYRZLCQQ-OELDTZBJSA-N | C00695 | 0.167 | -2.581 |  |
| Oxoglutaric acid | KPGXRSRHYNQIFN-UHFFFAOYSA-N | C00026 | 0.558 | -0.840 |  |
| Tyramine | DZGWFCGJZKJUFP-UHFFFAOYSA-N | C00483 | 1.277 | 0.353 |  |
| Phenylethylamine | BHHGXPLMPWCGHP-UHFFFAOYSA-N | C05332 | 0.052 | -4.257 |  |
| Glucosamine | MSWZFWKMSRAUBDIVMDWMLBSA-N | C00329 |  | 1.609 |  |
| D-Arabitol | HEBKCHPVOIAQTAQWWZWVQMSA-N | C01904 | 0.255 | -1.971 |  |
| Glycolic acid | AEMRFAOFKBGASWUHFFFAOYSA-N | C00160 | 0.352 | -1.502 |  |
| D-Threitol | UNXHWFMMPAWVPI-QWWZWVQMSA-N | C16884 | 0.255 | -1.971 |  |
| Cholesterol | HVYWMOMLDIMFJA-DPAQBDIFSA-N | C00187 | 1.207 | 0.153 |  |
| Myristic acid | TUNFSRHWOTWDNC-UHFFFAOYSA-N | C06424 | 0.730 | 0.177 |  |
| L-Lactic acid | JVTAAEKCZFNVCJ-REOHCLBHSA-N | C00186 | 0.844 | -0.243 |  |
| L-Glutamic acid | WHUUTDBJXJRKMK-VKHMYHEASA-N | C00025 | 0.988 | 0.155 |  |

(International Chemical Identifiers (InChI) and standard InChI hashes (InChIKey); KEGG = Kyoto Encyclopaedia of Genes and Genomes; FC = Fold change)

| **Pathway Name** | ***p* value** | **-log(p)** | **Holm p** | **FDR** | **Impact** | **Importance scores for compounds** |
| --- | --- | --- | --- | --- | --- | --- |
| Valine, leucine and isoleucine biosynthesis | 0.019846 | 3.9198 | 1 | 0.40684 | 0.28572 | Isoleucine: 0.142, Valine: 0.14, |
| Aminoacyl-tRNA biosynthesis | 0.0075182 | 4.8904 | 0.60146 | 0.2055 | 0.18 | Aspartic acid: 0.02, Serine: 0.04, Methionine: 0.02, Valine: 0.02, Isoleucine: 0.02 |
| Galactose metabolism | 0.0015514 | 6.4686 | 0.12567 | 0.063609 | 0.12904 | Melibiose: 0.06, Lactose: 0.064, Galactose: 0.06 |
| Glycine, serine and threonine metabolism | 0.1306 | 2.0356 | 1.0 | 0.97358 | 0.17143 | Threonine: 0.02, Serine: 0.1 |
| Cysteine and methionine metabolism. | 0.1036 | 2.2672 | 1.0 | 0.94388 | 0.11111 | Methionine: 0.037, Serine: 0.07, Cysteine: 0.11 |

**Table S3:** Results from ingenuity pathway analysis with MetPA in YC/YP

(The raw p is the original p value calculated from the MSEA analysis; the Impact is the pathway impact value calculated from pathway analysis; FDR= False discovery

rate Holm p = Holm-Bonferroni method)

**Table S4.** Results from metabolic pathway analysis using MetPA in AC/AP groups

| **Pathway Name** | **Match Status** | **p** | **-log(p)** | **Holm p** | **FDR** | **Impact** | **Importance scores for compounds** |
| --- | --- | --- | --- | --- | --- | --- | --- |
| D-Glutamine and D-glutamate metabolism | 2/5 | 0.0014908 | 6.5084 | 0.12225 | 0.12225 | 0.33333 | D-Glutamine: 0.33333 |
| Alanine, aspartate and glutamate metabolism | 2/24 | 0.035665 | 3.3336 | 1.0 | 0.73112 | 0.16 | Alanine: 0.04 aspartate: 0.04  Glutamate: 0.16 |
| Galactose metabolism | 3/26 | 0.003731 | 5.5911 | 0.30221 | 0.15297 | 0.12904 | Melibiose: 0.06452, Alpha-Lactose: 0.06452,  D-Galactose: 0.06452 |
| Nitrogen metabolism | 1/9 | 0.10898 | 2.2166 | 1.0 | 1.0 | 0.11111 | L-Glutamic acid: 0.11111, Ammonia: 0.33333 |
|  |  |  |  |  |  |  |  |
| Butanoate metabolism | 2/22 | 0.030301 | 3.4966 | 1.0 | 0.73112 | 0.10526 | L-Glutamic acid: 0.05263, Oxoglutaric acid: 0.05263 |

(The raw p is the original p value calculated from the MSEA analysis; the Impact is the pathway impact value calculated from pathway analysis; FDR=

False discovery rate Holm p = Holm-Bonferroni method)
