# Supplementary material for: Lactobacillus acidophilus DDS-1 Modulates the Gut Microbiota and Improves Metabolic Profiles in Aging Mice
Source: Nutrients. 2018 Sep 6;10(9):1255. doi: 10.3390/nu10091255 (PMC6165029; doi:10.3390/nu10091255)

**Figure S1**: Neighbour-net showcasing the three different clusters of all the samples using Brady-Curtis Ecological Index and Ward clustering (p < 0.01). (YC) Young control group, (YP) young probiotic group, (AC) aging control group, (AP) aging probiotic group.


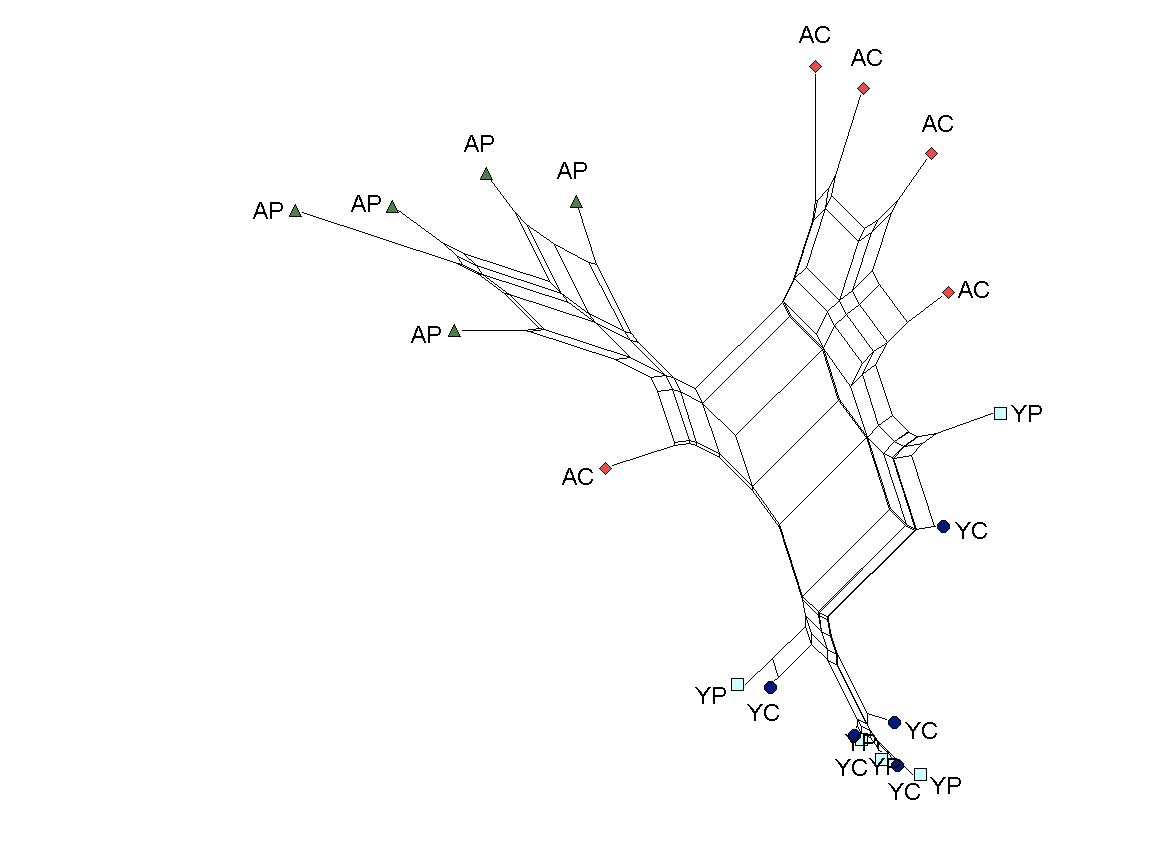


**Figure S2**. Heat map showing the Correlation analysis of fecal microbiota in aging and young groups at phylum level using Pearson r distance method. (p < 0.01)


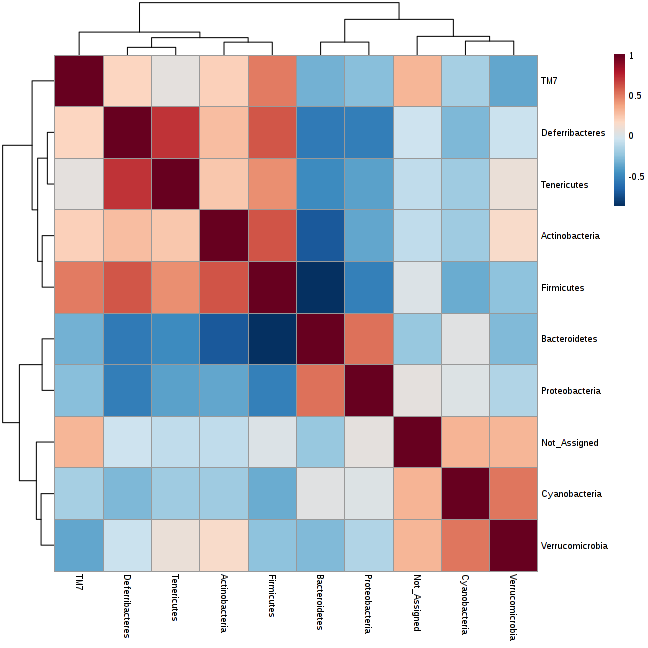


**Figure S3.** 2D O-PLS-DA plot with showing clear separation between YC and YP groups. (YC) Young control group, (YP) young probiotic group, (AC) aging control group, (AP) aging probiotic group.

A B


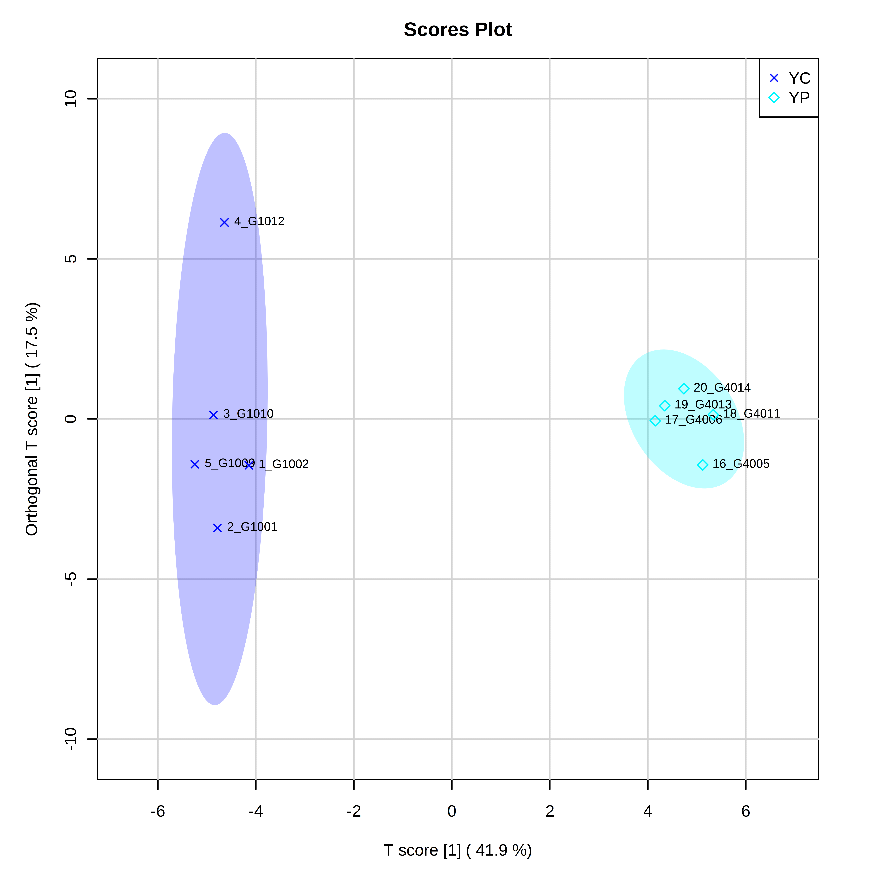

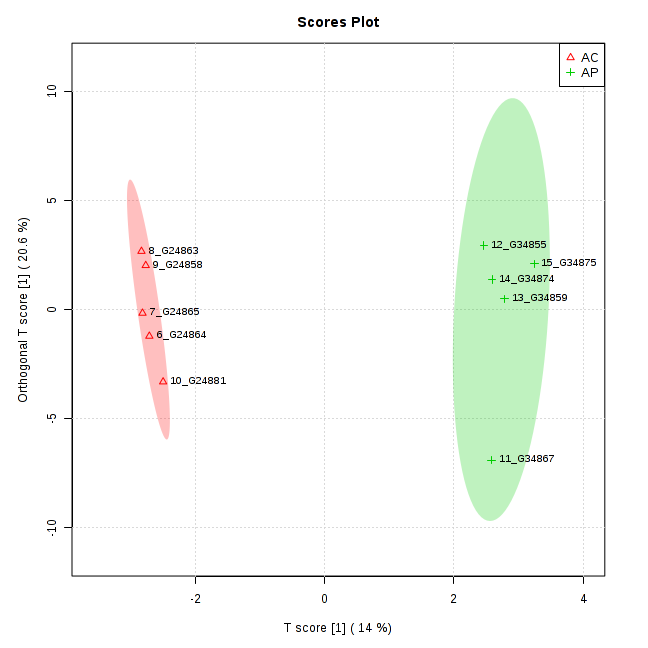


**Figure S4**. PCA plot of feces collected from YC and AC (**A**) and YP and AP (**C**) groups showing divergence. Key compounds separating YC and AC groups (**B**) based on variable importance in projection (VIP) score plot (**D**) in PLS-DA analysis. (YC) Young control group, (YP) young probiotic group, (AC) aging control group, (AP) aging probiotic group.

**A** **B**


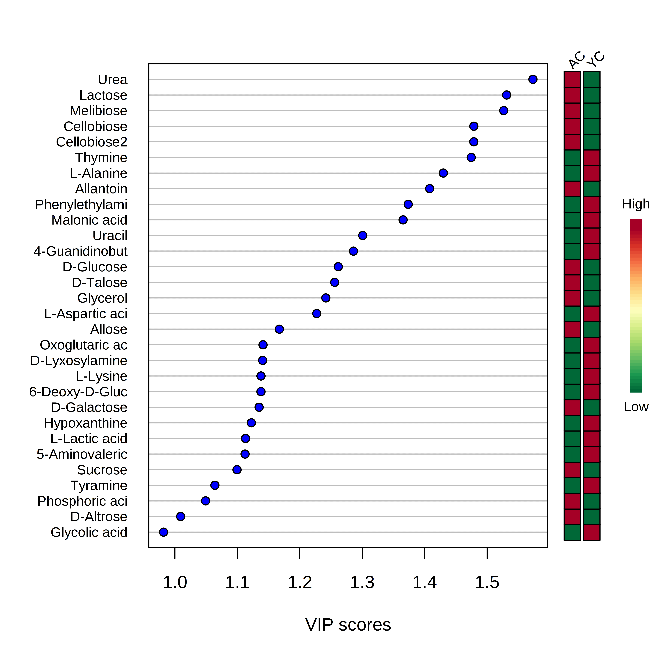

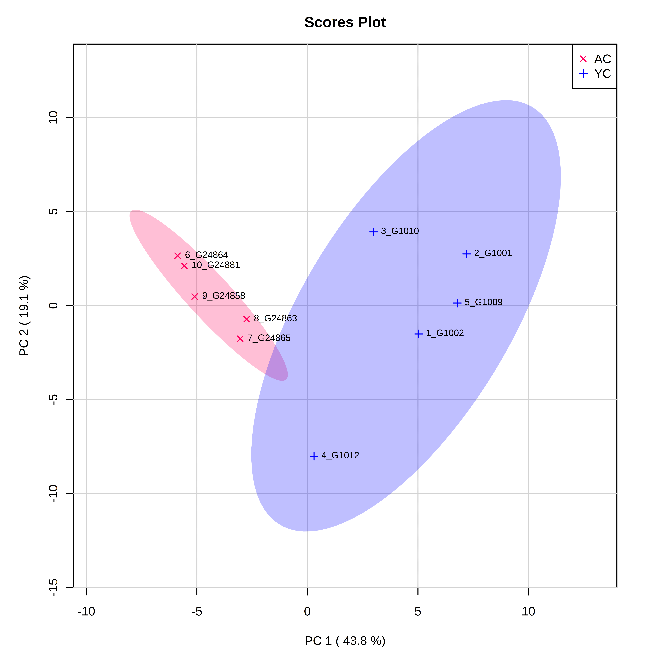


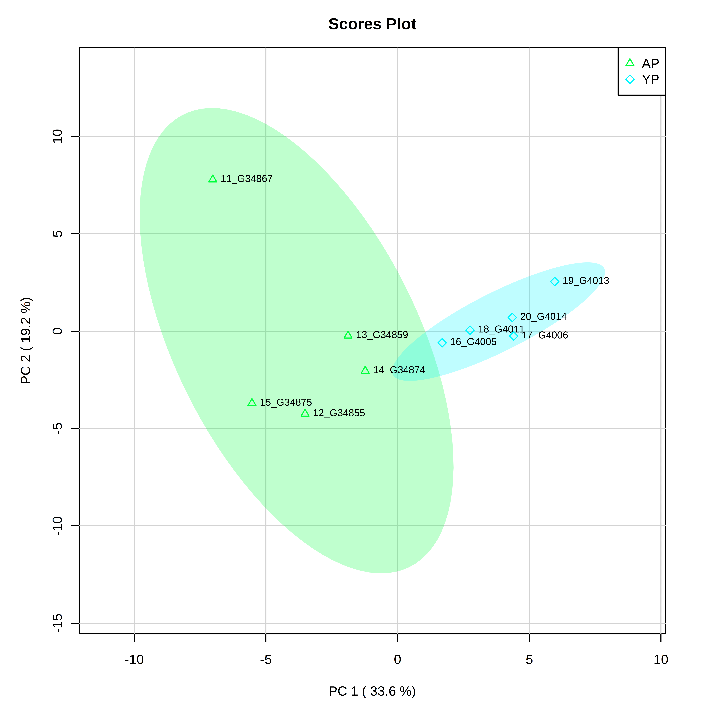
**C** **D**


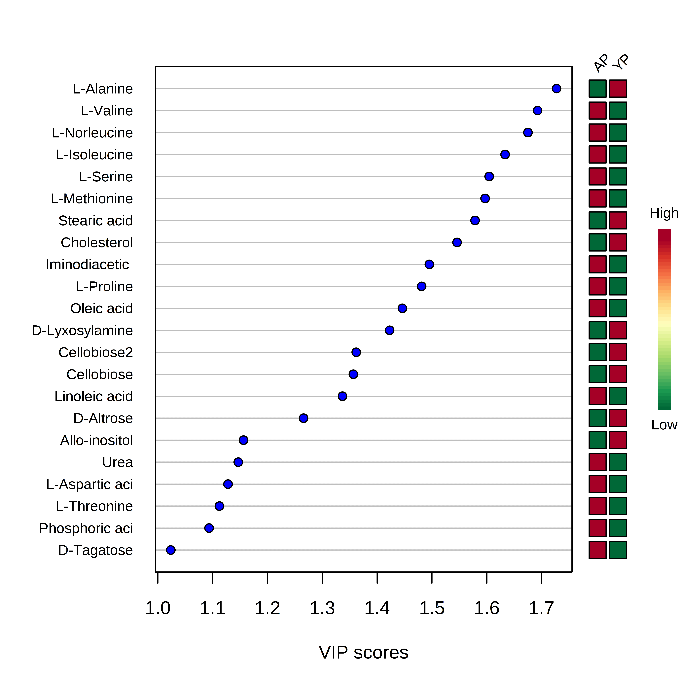


**Figure S4**. **A**. Bi-plot showing compounds responsible for divergence in young and aging groups **B**. Key compounds separating young and aging groups based on variable importance in projection (VIP) score plot in PLS-DA analysis.

**
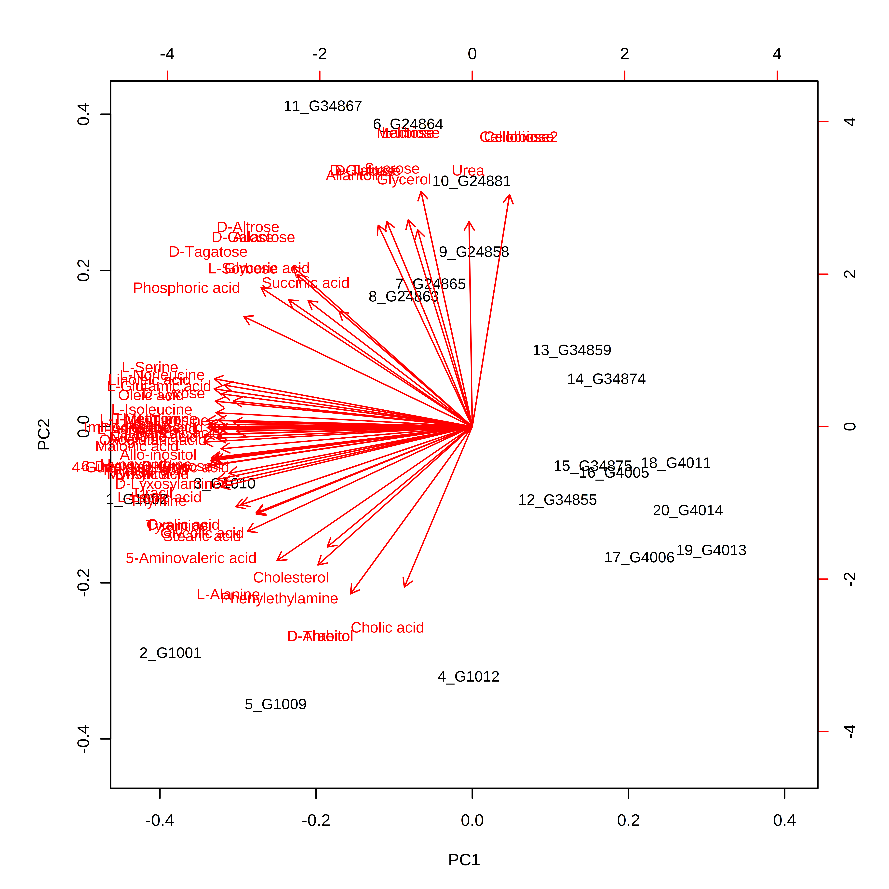

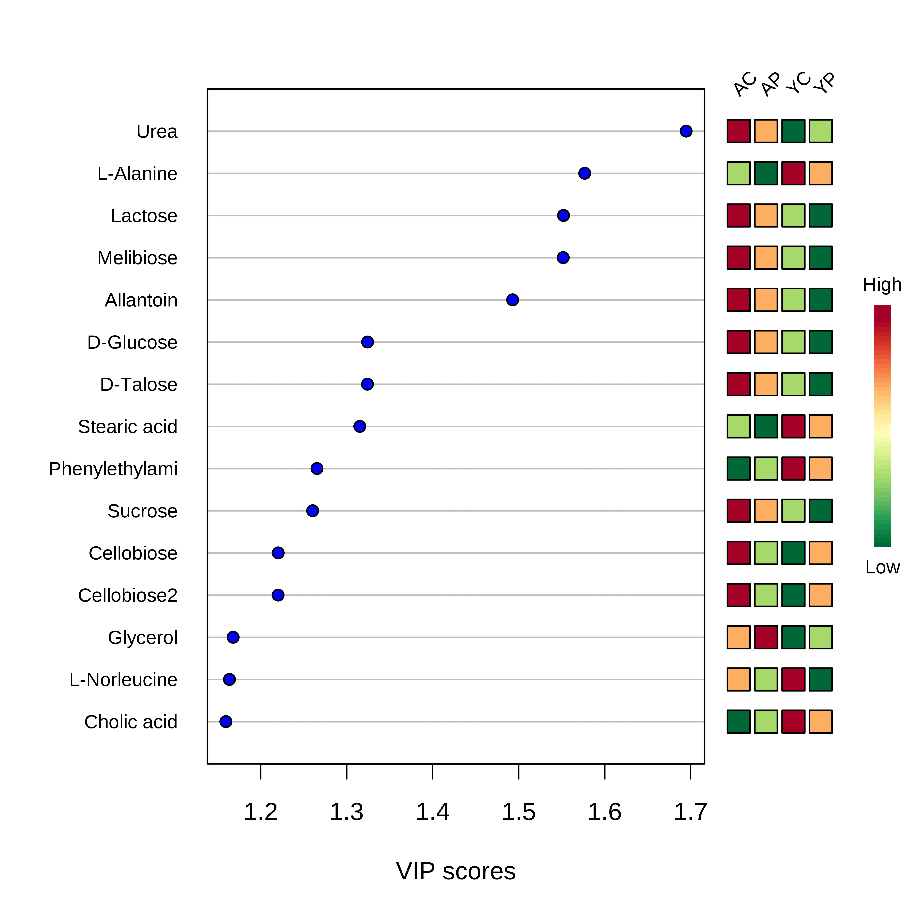
A** **B**

**Figure S6**. Significant changes in metabolites are expressed as heat map in all four groups. (YC) Young control group, (YP) young probiotic group, (AC) aging control group, (AP) aging probiotic group.


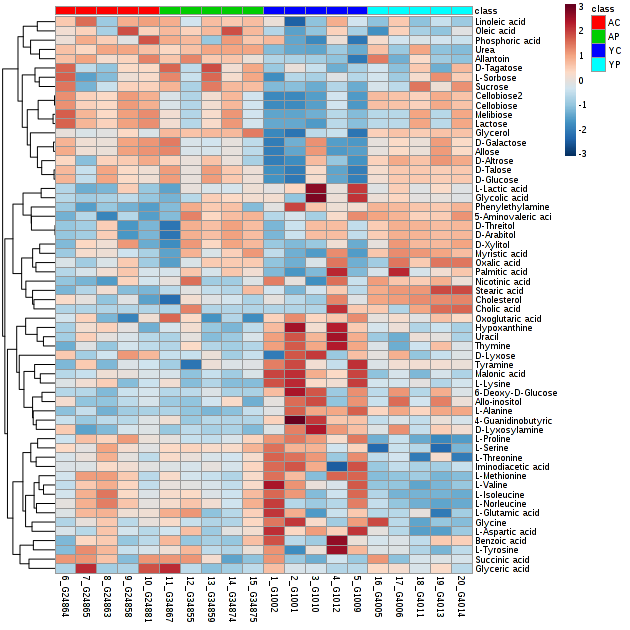


**Figure S7.** Overall metabolite cluster in YC, YP, AC and AP groups using Pearson’s R correlation with ward distancing method (p < 0.01). (YC) Young control group, (YP) young probiotic group, (AC) aging control group, (AP) aging probiotic group.

**
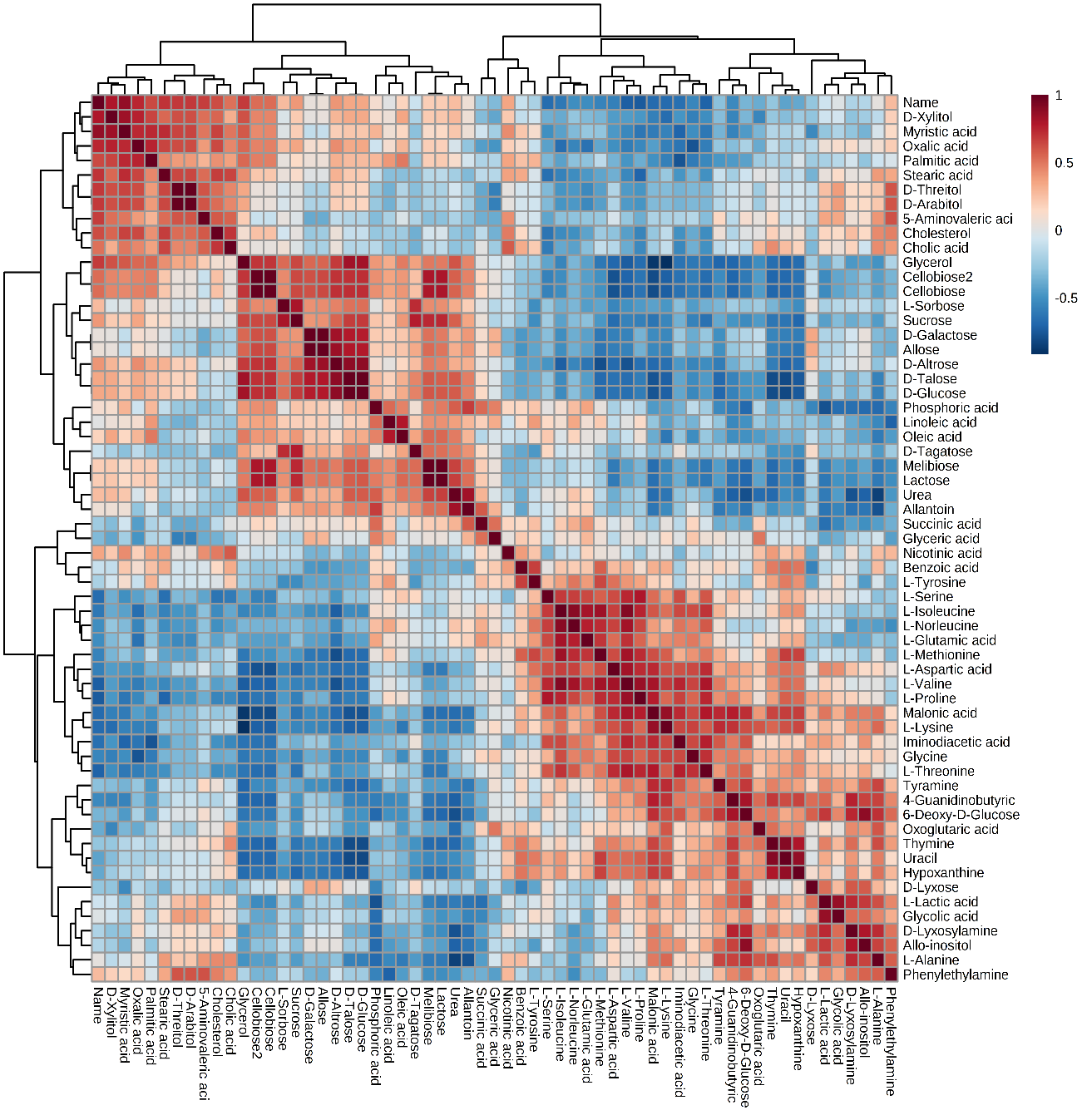
**

**Figure S8**. Age-wise and Group-wise correlation analysis showing the cluster of YP and AP and differences in YC and AC groups using Pearson’s correlation using ward distancing method (p < 0.01). (YC) Young control group, (YP) young probiotic group, (AC) aging control group, (AP) aging probiotic group.


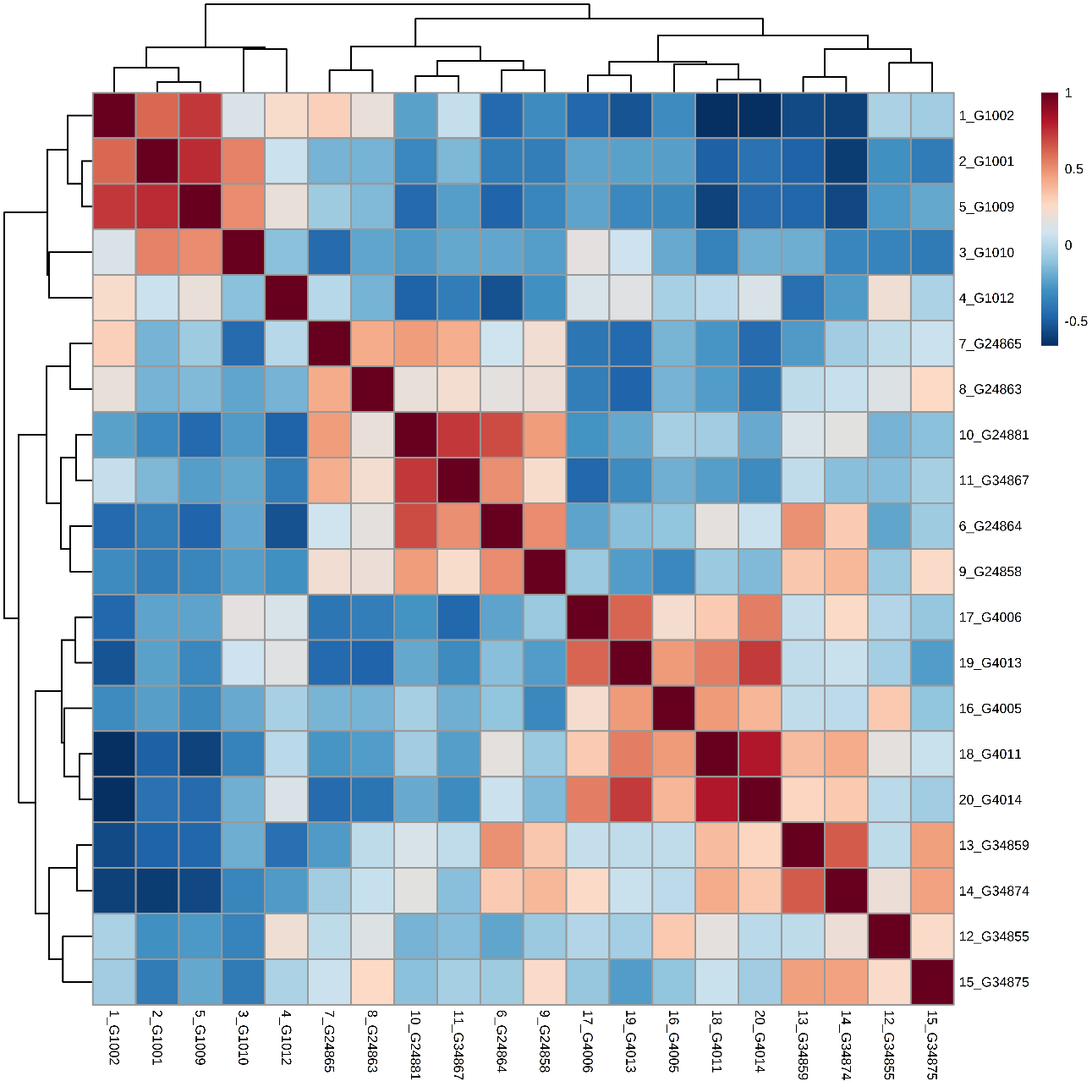


YC

AC

AP

YP

**Figure S9.** Potential pathways for Young probiotic (YP) from **Figure 8A** (**A**) Valine, leucine and isoleucine biosynthesis, (**B**) Glycine, serine and threonine metabolism, (**C**) Aminoacyl-tRNA biosynthesis. Compounds in red are significantly involved in the pathways**.**

**
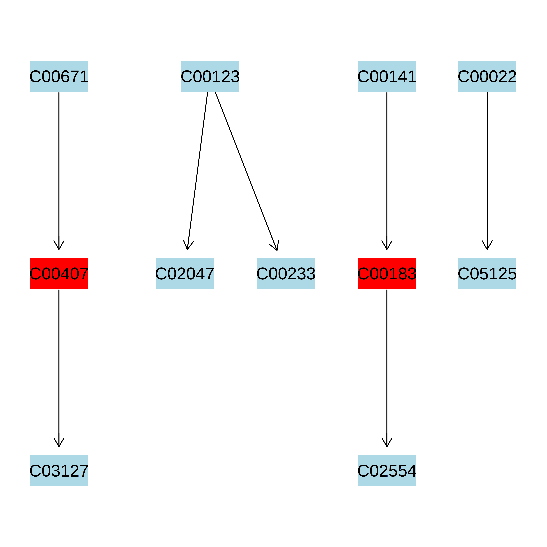
 A B**

**
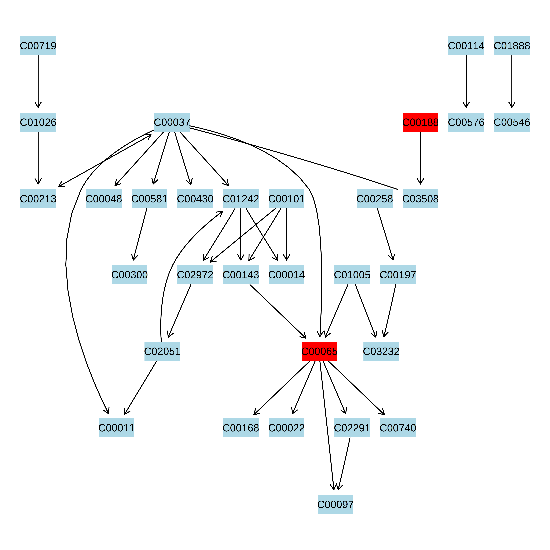
**

**
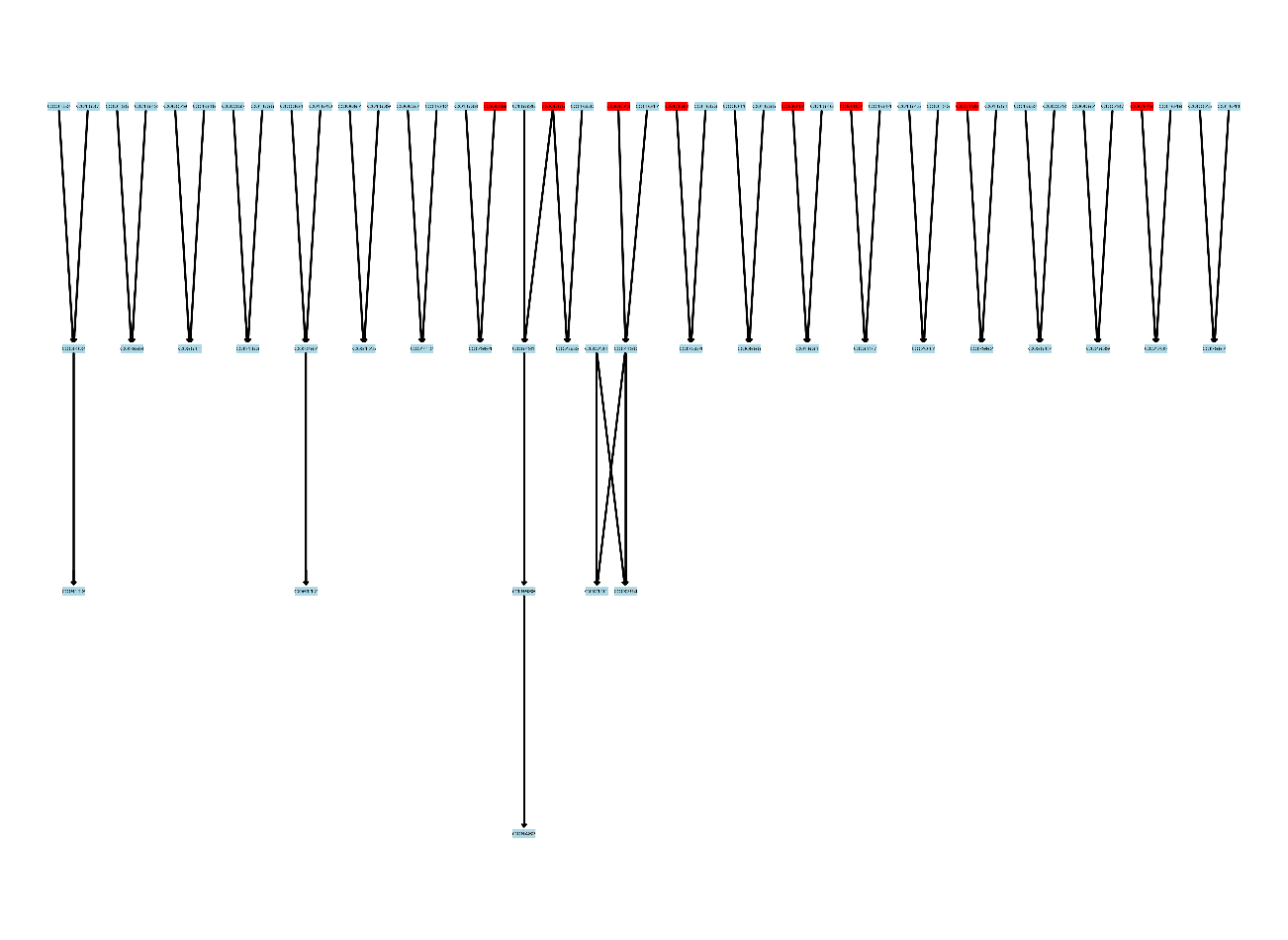
 C**

**Figure S10.** Potential pathways for Aging probiotic (AP) from **Figure 8B,** (**A**) D-Glutamine and D-glutamate metabolism (**B**) Alanine, aspartate and glutamate metabolism (**C**) Nitrogen metabolism, (**D**) Butanoate metabolism. Compounds in red are significantly involved in the pathways.

**A B**


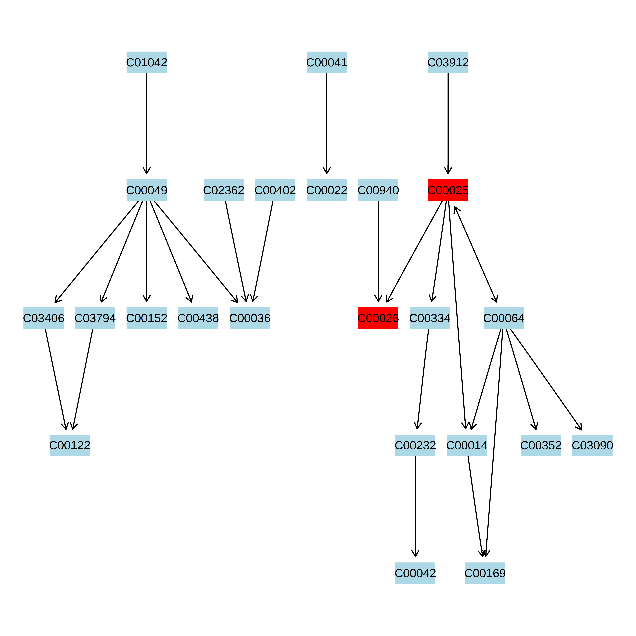

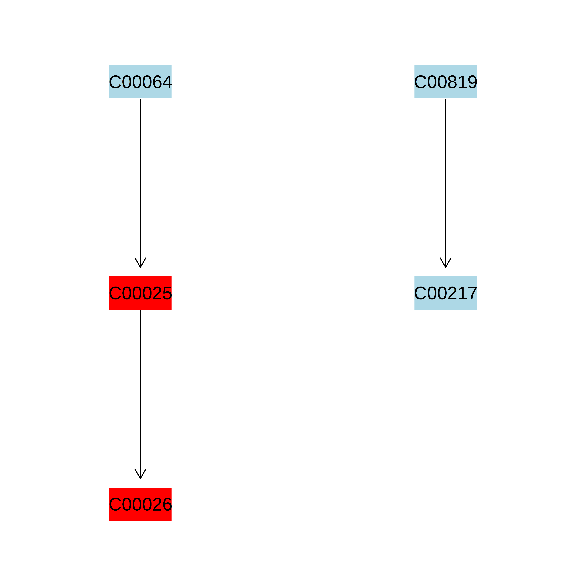
A


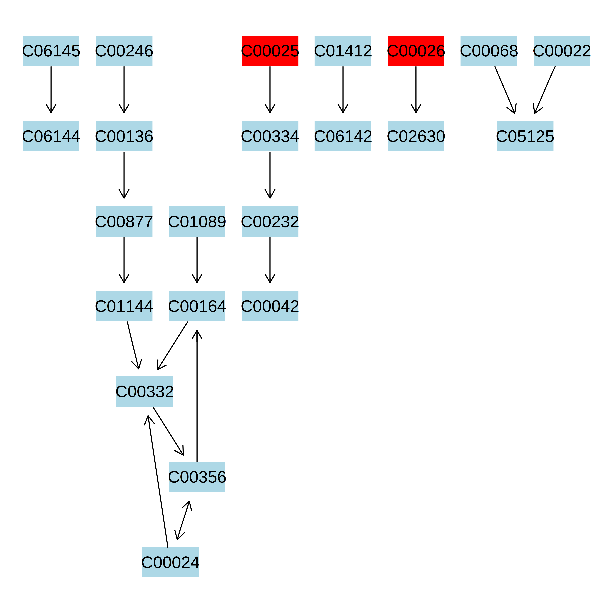
**C D**


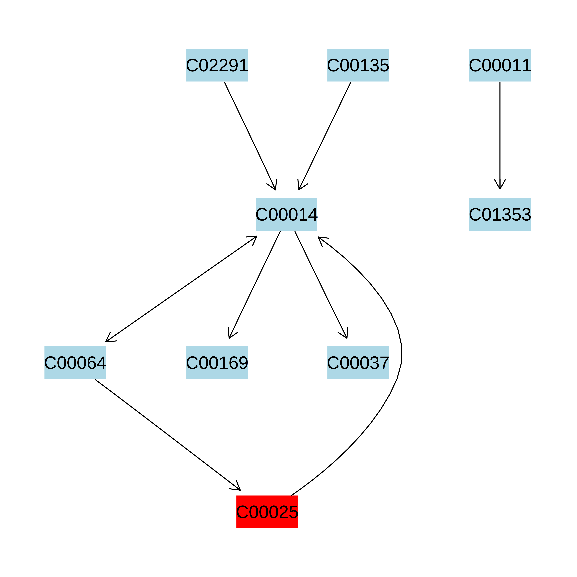


**Figure S11**. KEGGS pathways of Alanine, aspartate and glutamate metabolism (**A**) and Galactose Metabolism (**B**). Compounds highlighted in red and green are directly involved in respective pathways.

**A**


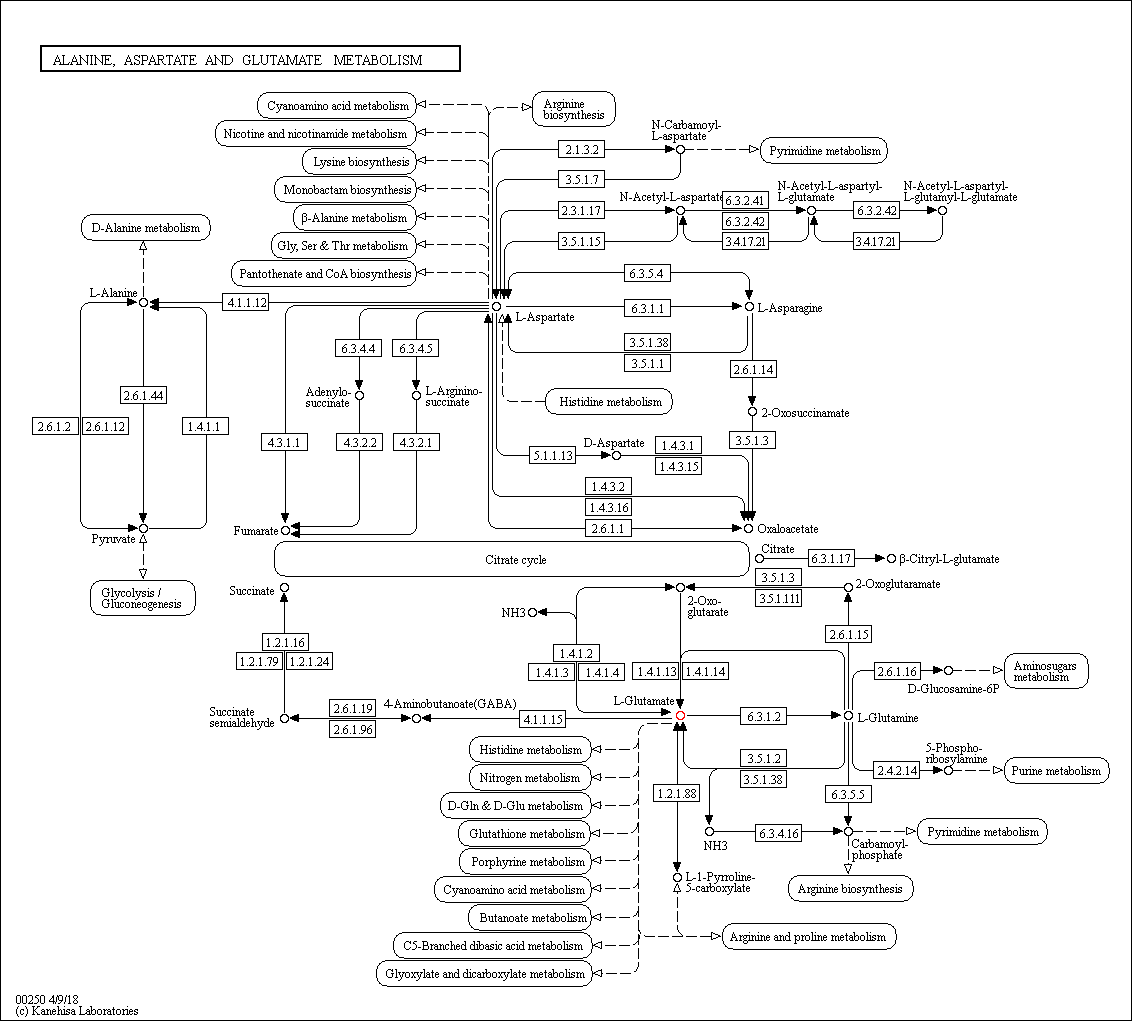


**B**


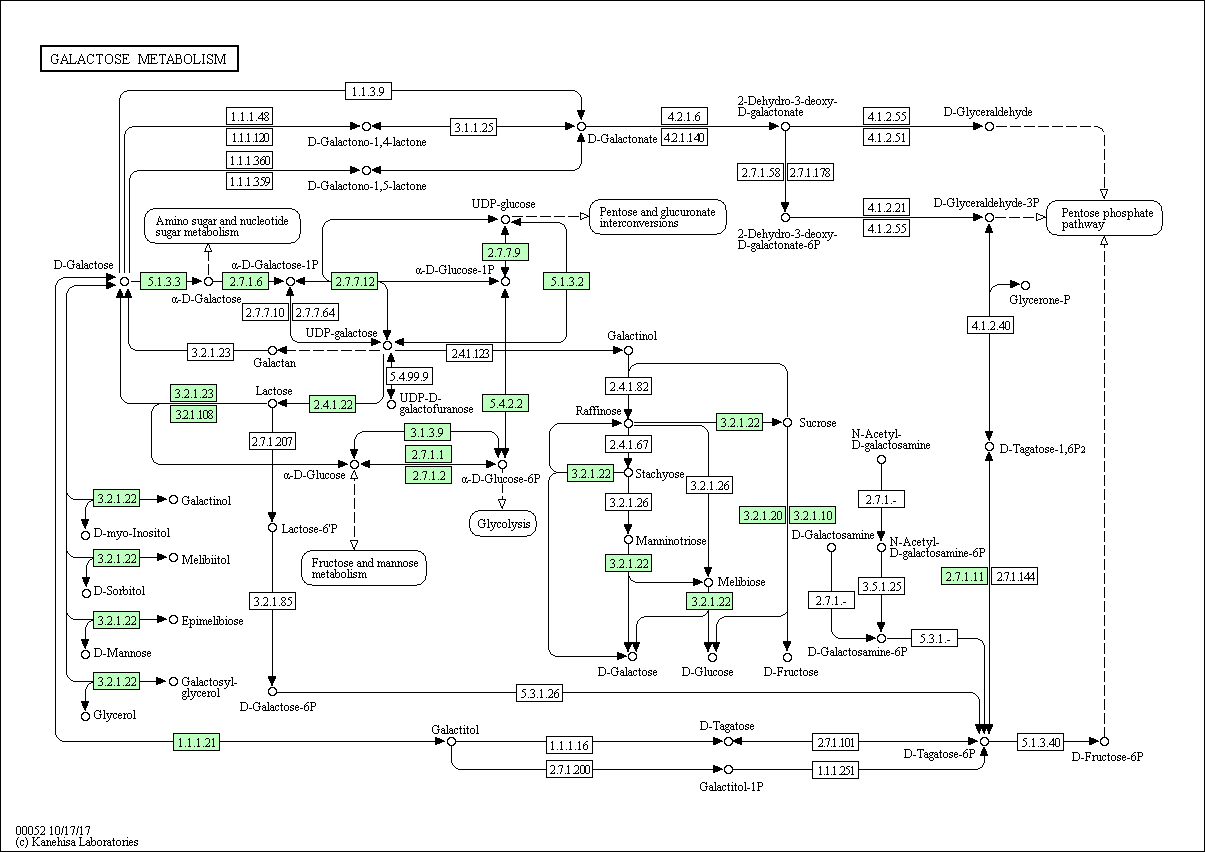


**Figure S12**. KEGGS pathways of Butanoate metabolism (**A**) and Nitrogen Metabolism (**B**). Compounds highlighted in red box and green are directly involved in respective pathways.

**A**


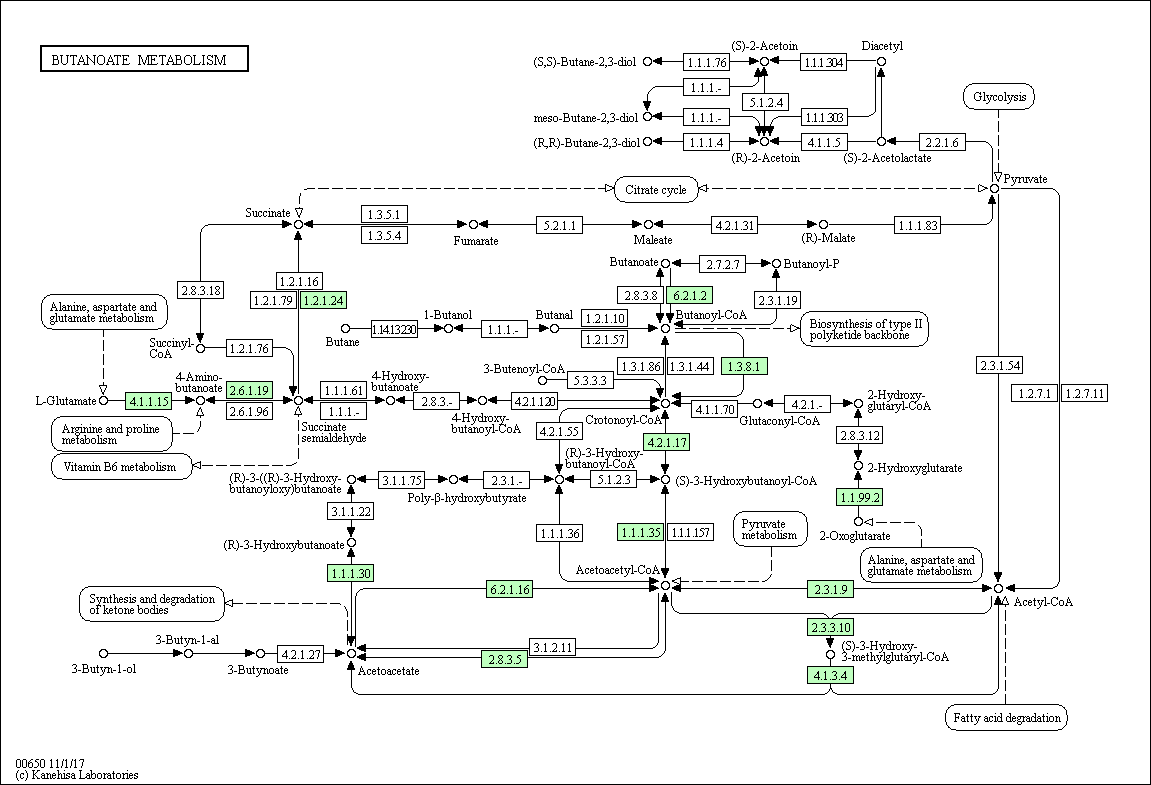


**B**


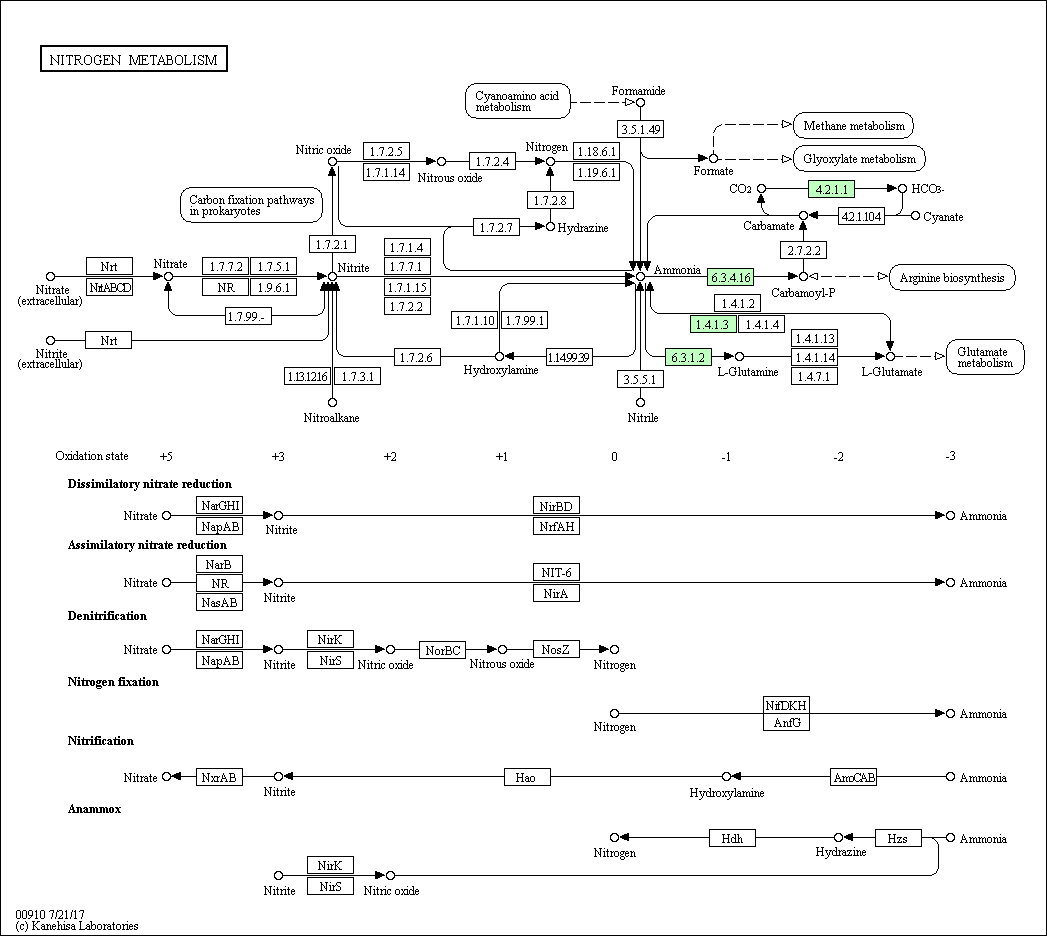


**Figure S13**. KEGGS pathways of Aminoacyl-tRNA biosynthesis. Compounds highlighted in red are directly involved in respective pathways.


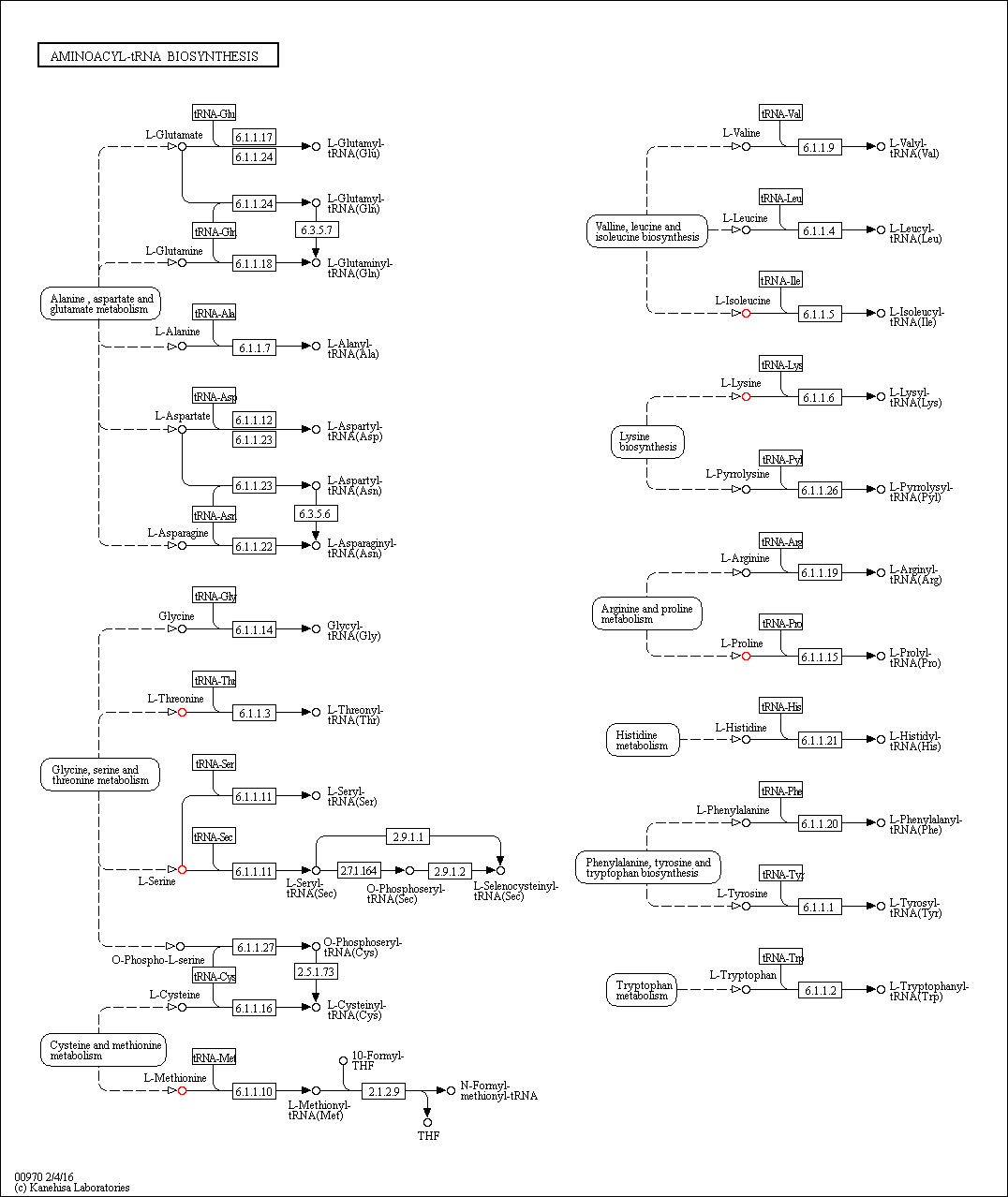

Supplement: Supplementary file 1 [file nutrients-10-01255-s001.zip › Supplemtary Figures.docx]
